# Highly potent and selective PPARδ agonist reverses memory deficits in mouse models of Alzheimer's disease

**DOI:** 10.7150/thno.96707

**Published:** 2024-09-16

**Authors:** Hyeon Jeong Kim, Haelee Kim, Jaeyoung Song, Jun Young Hong, Elijah Hwejin Lee, Ashwini M. Londhe, Ji Won Choi, Sun Jun Park, Eunseok Oh, Heeseok Yoon, Hoosang Hwang, Dongyup Hahn, Kyungjin Jung, Sugyeong Kwon, Tara Man Kadayat, Min Jung Ma, Jeongmin Joo, Jina Kim, Jae Hyun Bae, Hayoung Hwang, Ae Nim Pae, Sung Jin Cho, Jong-Hyun Park, Jungwook Chin, Heonjoong Kang, Ki Duk Park

**Affiliations:** 1 Center for Brain Disorders, Korea Institute of Science and Technology (KIST), Seoul 02792, Republic of Korea.; 2 New Drug Development Center, Daegu-Gyeongbuk Medical Innovation Foundation, Daegu 41061, Republic of Korea.; 3 Laboratory of Marine Drugs, School of Earth and Environmental Sciences, Seoul National University, NS-80 Seoul 08826, Republic of Korea.; 4 Department of Systems Biology, Yonsei University, Seoul 03722, Republic of Korea.; 5 Division of Bio-Medical Science & Technology, KIST School, Korea University of Science and Technology, Seoul 02792, Republic of Korea.; 6 Cureverse, lnc., H2 building, KIST, Seoul 02792, Republic of Korea.; 7 School of Food Science and Biotechnology, Kyungpook National University, Daegu 41566, Republic of Korea.; 8 Research Institute of Oceanography, Seoul National University, NS-80, Seoul 08826, Republic of Korea.

**Keywords:** PPARδ agonist, Alzheimer's disease, anti-inflammation, glial activation, BACE1

## Abstract

**Rationale:** Alzheimer's disease (AD) is a progressive neurodegenerative disease accompanied by neurotoxicity, excessive inflammation, and cognitive impairment. The peroxisome proliferator-activated receptor (PPAR) δ is a potential target for AD. However, its regulatory mechanisms and therapeutic potential in AD remain unclear. We aimed to investigate if the activation of PPARδ using a highly selective and potent agonist could provide an effective therapeutic strategy against AD.

**Methods:** We synthesized a novel PPARδ agonist, **5a,** containing a selenazole group and determined the X-ray crystal structure of its complex with PPARδ. The drug-like properties of **5a** were assessed by analyzing cytochrome P450 (CYP) inhibition, microsomal stability, pharmacokinetics, and mutagenicity. We investigated the anti-inflammatory effects of **5a** using lipopolysaccharide (LPS)-stimulated BV-2 microglia and neuroinflammatory mouse model. The therapeutic efficacy of **5a** was evaluated in AD mice with scopolamine-induced memory impairment and APP/PS1 by analyzing cognitive function, glial reactivity, and amyloid pathology.

**Results:** Compound **5a**, the most potent and selective PPARδ agonist, was confirmed to bind hPPARδ in a complex by X-ray crystallographic analysis. PPARδ activation using **5a** showed potent anti-inflammatory effects in activated glial cells and mouse model of neuroinflammation. Administration of **5a** inhibited amyloid plaque deposition by suppressing the expression of neuronal beta-site amyloid precursor protein cleaving enzyme 1 (BACE1), and reduced abnormal glial hyperactivation and inflammatory responses, resulting in improved learning and memory in the APP/PS1 mouse model of AD.

**Conclusion:** We identified that specific activation of PPARδ provides therapeutic effects on multiple pathogenic phenotypes of AD, including neuroinflammation and amyloid deposition. Our findings suggest the potential of PPARδ as a promising drug target for treating AD.

## Introduction

Peroxisome proliferator-activated receptors (PPARs) are nuclear transcriptional factors belonging to the ligand-activated nuclear receptor superfamily and consist of three known subtypes: PPARα, γ, and δ 1,2. Upon activation, these receptors heterodimerize with retinoid X receptor and regulate the expression of genes involved in energy utilization, cell differentiation, proliferation, mitochondrial function, glucose homeostasis, and lipid metabolism 3-5. Thus, PPARs have been mainly studied in muscle or peripheral organs as therapeutic targets for drugs against cancer, cardiovascular disease, and metabolic disorders such as diabetes 5-7.

PPARδ, also known as PPARβ, is ubiquitously expressed in murine and human tissues, being more abundant in the brain than other PPAR subtypes 8-10. PPARδ is expressed in most brain cell types, including neurons, astrocytes, and microglia 11-13, where it regulates neurogenesis, oxidative stress, neuroinflammation, as well as lipid metabolism 14-18. In mouse models of neurodegenerative disease or ischemic stroke, PPARδ agonists suppress neuronal cell loss 19,20, mitochondrial dysfunction 20, activation of nuclear factor (NF)-κB or nucleotide-binding domain and leucine-rich-repeat-protein 3 (NLRP3) inflammasome 21,22, and neutrophil infiltration 23, 24 and reduce the expression of several pro-inflammatory chemokines and cytokines such as tumor necrosis factor (TNF)-α and interleukin (IL)-6 21, 24. These results indicate that PPARδ agonists exert potent neuroprotective and anti-inflammatory effects in the central nervous system (CNS). PPARδ-null mice and transgenic mice expressing dominant-negative PPARδ in neurons exhibit fatal defects such as brain atrophy, myelin alternation, impaired brain development, and widespread neurodegeneration 19, 25, 26. Despite its obvious pivotal role in the CNS, the underlying protective mechanism of PPARδ against neurodegeneration and neuroinflammation remains elusive.

Alzheimer's disease (AD) is the most frequent neurodegenerative disease and is characterized by pathological features such as the deposition of amyloid β (Aβ) plaques and neurofibrillary tangles and clinical symptoms such as progressive memory loss and cognitive decline 27. Excessive neuroinflammation, accompanied by hyperactivation of glial cells and prolonged release of inflammatory mediators, is considered an early key event exacerbating the pathogenesis and progression of AD 28, 29. The level of PPARδ expression is significantly lower in the brains of patients with AD than in those of the general population. Similar results are observed in mice with AD compared to their non-AD counterparts 30, 31. Genetic deletion of PPARδ leads to cognitive impairment with increased levels of enzymes involved in Aβ deposition, tau hyperphosphorylation, neuroinflammation, and astrogliosis 32. In mouse models of AD, PPARδ activation using specific agonists reverses the pathological manifestations of AD through anti-inflammatory effects 31, 33-37. Although no FDA-approved drugs specifically target PPARδ, a recent exploratory phase Ⅱa clinical trial has reported that the dual PPARδ/γ agonist T3D-959 improves cognitive function in patients with mild-to-moderate AD 35, 38. However, long-term use of PPARγ-activating drugs was associated with side effects 39, 40. Therefore, highly selective PPARδ agonists are desirable as potential therapeutic agents in AD.

A representative potent and selective PPARδ agonist that has been developed is GW501516. GlaxoSmithKline discovered and evaluated GW501516 as a drug candidate for treating metabolic and cardiovascular diseases 6. However, owing to concerns about side effects related to its PPARγ-activating efficacy, the clinical trial was discontinued 40, 41. Based on these data, we first synthesized various derivatives of GW501516 to develop a highly potent and selective PPARδ agonist. Among them, the PPARδ agonist **5a** containing selenazole core showed excellent agonistic effect and selectivity for PPARδ. We studied the structure of the complex **5a**-hPPARδ using X-ray crystallographic analysis and further investigated the therapeutic effects of **5a** on neuroinflammation and AD-like pathology using cellular and mouse models.

## Results

### Synthesis of novel PPARδ agonists with a selenazole core

**Figure 1:**
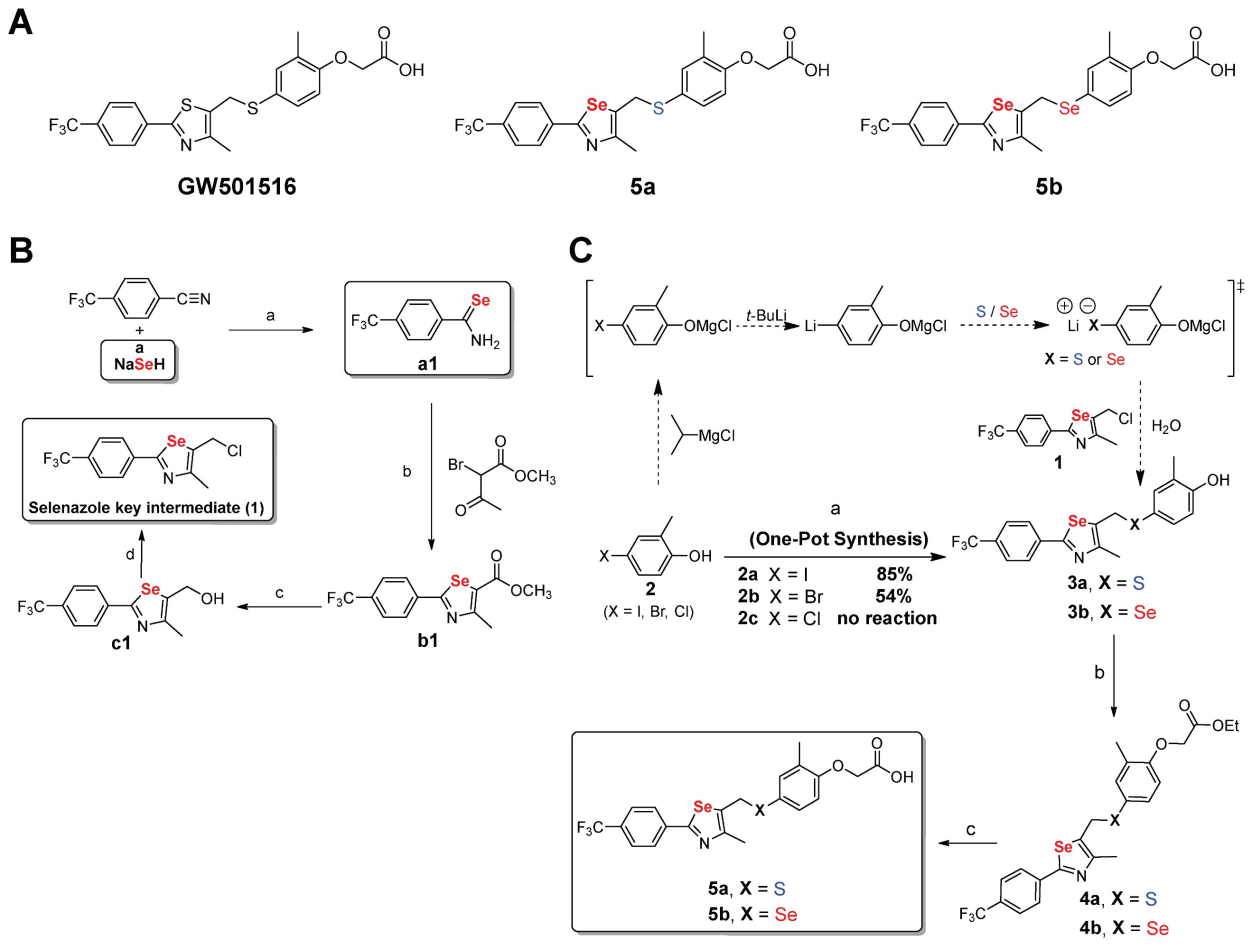
** Synthesis of novel PPARδ agonists containing one selenazole moiety. (A)** Structures of GW501516 and PPARδ agonists with a selenazole core (**5a** and **5b**). **(B)** Synthesis of key selenazole intermediate. Reagents and conditions: (a) The reaction between HCl 2 M and pyridine/ethanol occurred for 1 h at a temperature varying from room temperature (r.t.) to 100 ^o^C (84%); (b) methyl 2-chloroacetoacetate and tetrahydrofuran (THF) reacted for 12 h at a temperature varying from r.t. to 80 ^o^C (95%); (c) diisobutyl aluminum hydride (DIBAL-H) and dichloromethane (DCM) reacted for 1 h at a temperature varying from -78 to -10 ^o^C (94%); and (d) triphenylphosphine (TPP), DCM, and *N*-chlorosuccinimide (NCS) reacted at r.t. for 10 h (90%). **(C)** Synthesis of selenium-containing PPARδ agonists. Reagents and conditions: (a) (i) Isopropylmagnesium chloride (1.0 equiv) and THF reacted at 0 ^o^C for 10 min; (ii) *t*-butyllithium (2.0 equiv) was added at -78 ^o^C for 0.5 h; (iii) sulfur powder (for **3a**) or selenium powder (for **3b**) and THF reacted at -78 to -20 ^o^C for 1 h; (iv) selenazole key intermediate (**1**) and THF reacted at 0 ^o^C for 0.5 h (85%); (b) Ethyl bromoacetate, K_2_CO_3_ and aq. acetone reacted at r.t. for 4 h (~95%); (c) (i) 2.0 M LiOH and aq. THF reacted at r.t. for 1 h; (ii) the product was acidified with 0.5 M NaHSO_4_ (90-92%).

To develop a highly potent and selective PPARδ agonist, we synthesized various derivatives of GW501516 by introducing a selenium-containing heterocyclic core (Figure 1A). We first optimized the synthesis method for incorporating selenium-containing heterocycles (selenazoles). During this process, we identified that selenocarboxamides played a crucial role in the formation of key intermediates. Specifically, we observed that nitriles react with sodium hydrogen selenide, pyridine, and hydrochloric acid in ethanol, forming primary aryl and alkyl selenocarboxamides 42. The aryl selenocarboxamide is converted into selenazole following its reaction with methyl-2-chloroacetoacetate. We first attempted to prepare the intermediate sulfide/selenide (**3a**, **3b**) from 4-bromo- or 4-iodo-2-methyl phenol **2** as a starting material using the one-pot method that included a four-step reaction: protection of the phenol moiety, lithium-halogen exchange, sulfur/selenium insertion, and quenching of the reaction by adding selenazole (Figure 1B-C).

Starting materials (**2a**-**c**) were commercially available or easily prepared from *o*-cresol using general halogenation methods. We found that *in-situ* conversion of the phenol group of starting material **2** using iPrMgCl (transforming -OH to -O MgCl) offered protection during the lithium-halogen exchange and sulfur/selenium insertion steps. We chose the one-pot method instead of a step-by-step method, which led to a good yield of the intermediate **3**. In the second step, treatment of intermediate **3** with K_2_CO_3_ and ethyl bromoacetate in aqueous acetone at room temperature (20-25 °C) for 4 h resulted in ester **4** with high yield (~95%) (Figure 1C). For the final step, hydrolysis of the ethyl ester **4** with 2M LiOH led to compounds **5a** and **5b**, with yields of 92 and 90%, respectively.

The half maximal effective concentration (EC_50_) values for compounds **5a** and **5b** were derived from an *in vitro* co-transfection assay assessing their affinity and activity towards human PPAR subtypes (Table 1). Our synthesized PPARδ agonist **5a** exhibited sub-nanomolar potency (EC_50_ = 0.7 nM) and excellent selectivity for hPPARδ (>14,000-fold selectivity over hPPARα and hPPARγ), demonstrating similar efficacy and better selectivity compared to GW501516 (EC_50_ = 1.2 nM and 250-fold selectivity over hPPARα; 1,000-fold selectivity over hPPARγ) (Table 1).

### Molecular docking study and X-ray crystallographic analysis of the ligand-human-PPARδ complexes

Batista *et al.* reported the crystal structure of the PPARδ-ligand complex, suggesting that VAL312 and ILE328 in the buried hormone-binding pocket play a crucial role in PPARδ selective binding. Notably, VAL312 and ILE328 in PPARδ have shorter side chain residues than those in PPARα (ILE339 and MET355) and PPARγ (MET348 and MET364) 43.

To explore the binding poses and molecular interactions of compounds **5a** and **5b**, we conducted molecular docking studies. Compounds **5a**, **5b,** and GW501516 exhibited higher binding energy in PPARδ compared to PPARα and PPARγ (Table S1) and showed similar interactions inside the PPARδ binding site (Figure S1). We subsequently superimposed the binding poses of **5a** inside PPARδ, PPARγ, and PPARα (Figure S2). In the **5a**-PPARδ complex (magenta), the presence of short residues (VAL312 and ILE238) allows the molecules to fit snugly within the ligand-binding pocket, facilitating a suitable conformation for hydrogen bonding with HIS413, TYR437, and HIS287. Conversely, the long side chains at the corresponding positions in PPARα and PPARγ hinder **5a** from adopting a conformation that can form hydrogen bonds with these critical residues (Figure S2).

**Figure 2:**
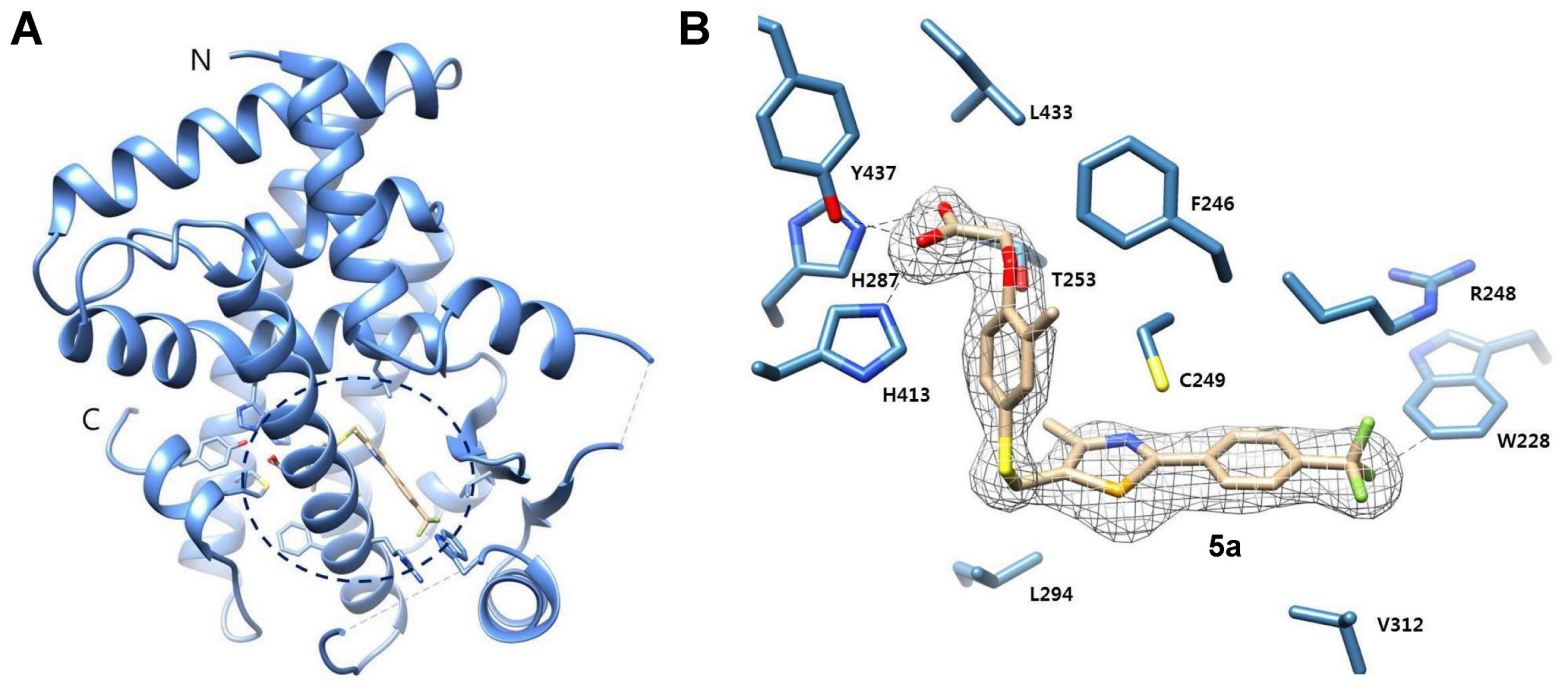
** X-ray crystal structure of 5a bound to the ligand-binding domain (LBD) of human PPARδ (171 - 441). (A)** The overall folding of the hPPARδ-**5a** complex structure. Residues 204-206 and 229-234 are missing due to insufficient electron density. **(B)** Protein residues are represented in blue, while **5a** is designated with beige. An Fo-Fc omit electron density map, contoured at 3.0 σ, is depicted, with the bound ligand shown as a grey mesh. Sulfur is displayed in yellow, selenium in dark yellow, nitrogen in dark blue, oxygen in red, and fluoride in green (PDB code: 5Y7X).

To clearly understand the binding mode of **5a** in the ligand-binding site, we determined the crystal structure of the ligand-binding domain (LBD) in the complex **5a**-hPPARδ (171-441) (Figure 2). Compound **5a** binds in the ligand-binding pocket in a similar manner to GW501516 39. The acetic acid moiety forms hydrogen bonds with the side chains of H287, H413, and Y437; corresponding bond lengths are 2.8, 2.7, and 2.6 Å, respectively. A pi-sulfur interaction with a bond length of 3.9 Å was observed between the selenazole ring in **5a** and the C249 side chain.

Hydrophobic interactions with side chains of residues L433, T253, F246, L294, V312, and R248 stabilize the complex receptor-**5a**. The trifluoromethyl group in **5a** establishes a weak hydrogen bond (2.9 Å) with the side chain of W228. This interaction was not observed in the co-crystal structure of the complex GW501516-hPPARδ, which showed a different rotameric conformation of the W228 indole group 39. The increased potency of **5a** compared to that of GW501516 for hPPARδ is likely attributable to its additional contacts with the W228 indole ring through a hydrogen bond involving C(sp3)-F-H-C (sp2).

### *In vitro* absorption, distribution, metabolism, excretion, and toxicity (ADMET) and *in vivo* pharmacokinetic profiles

The drug-like properties of compound **5a** were subsequently measured using a cytochrome P450 (CYP) inhibition test, liver microsomal metabolic stability test, and human Ether-à-go-go related gene (hERG) assay. To identify potential drug-drug interactions, we examined the inhibitory effects of **5a** on five CYP isotypes: 1A2, 2C9, 2C19, 2D6, and 3A4. Compound **5a** exhibited low inhibitory activities against CYP subtypes at 10 µM (IC_50_ > 10 µM) (Table S2). It showed excellent metabolic stability in human liver microsomes and low inhibitory effects on the hERG channel (Table S2). We also investigated the *in vivo* pharmacokinetic (PK) properties of compound **5a** after intravenous injection (*i.v.*) of a dose of 1 mg/kg or oral administration (*p.o.*) of a dose of 10 mg/kg in rats (Table S3). Compound **5a** showed high oral bioavailability (*F* = 90.7 %) with an exceptional AUC value (13331 hr*ng/mL), a substantially high *C*_max_ value (2744.3 ng/mL), and low plasma clearance (*CL* = 10.2 mL/min/kg).

Next, the mutagenic potential of **5a** was evaluated using a two-strain Ames test, in which *Salmonella typhimurium* strains TA98 and TA100 were used to detect frameshift and base substitution mutations, respectively. Mutagenicity testing indicated that **5a** lacks mutagenicity in the absence as well as in the presence of the S9 fraction in both TA98 and TA100 strains (Table S4). Altogether, these results indicate excellent PK profiles in rats after both *i.v.* and *p.o.* administration and favorable drug-like properties of **5a**.

### Compound 5a suppresses inflammatory responses in activated glial cells *in vitro*

**Figure 3:**
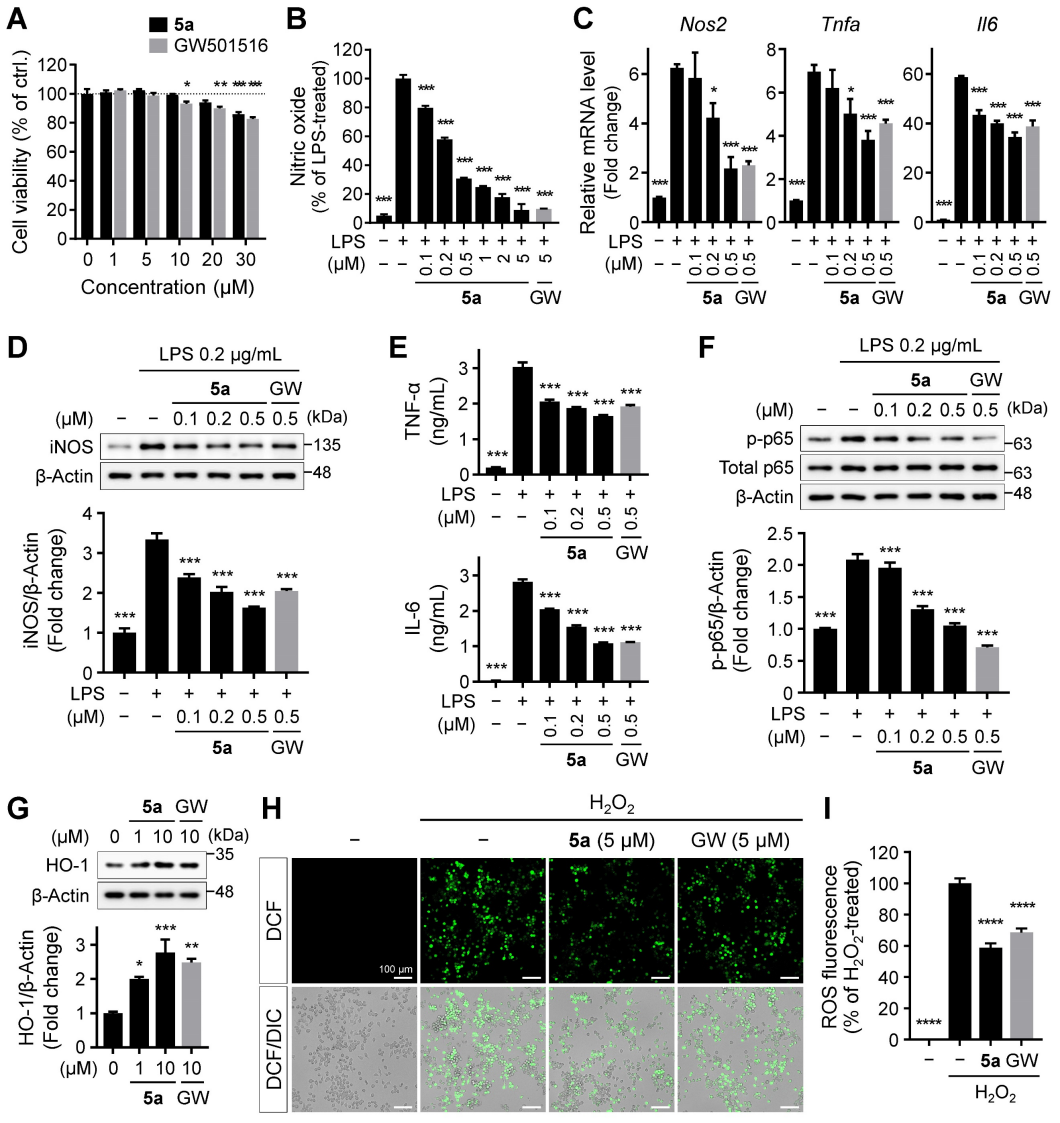
** 5a reduces LPS-induced inflammatory responses in BV-2 microglia. (A)** Viability of BV-2 microglial cells after treatment with **5a** or GW501516 for 24 h. *P < 0.05, **P < 0.01 and ***P < 0.001 versus vehicle-treated control (one-way ANOVA with Dunnett's test). **(B)** Nitrite levels in the culture medium of BV-2 cells pretreated with **5a** or GW501516 for 3 h followed by LPS (0.2 μg/mL) treatment for 24 h. **(C)** qRT-PCR analysis of relative mRNA expression of *Nos2* (*iNos*), *Tnfa*, and *Il6* in BV-2 cells pretreated with **5a** or GW501516 for 12 h followed by LPS treatment for 3 h. *Hprt* mRNA levels were used to normalize the expression of each gene. **(D)** Western blot analysis for iNOS in BV-2 cells pretreated with **5a** or GW501516 for 1 h followed by LPS treatment for 24 h. **(E)** TNF-α and IL-6 levels in the culture medium of BV-2 cells pretreated with **5a** or GW501516 for 9 h followed by LPS treatment for 18 h. **(F)** Western blot analysis for phospho-NF-κB p65 (Ser536) and NF-κB p65 in BV-2 cells pretreated with **5a** or GW501516 for 6 h followed by LPS treatment for 1 h. *P < 0.05 and ***P < 0.001 versus LPS-treated control (one-way ANOVA with Dunnett's test). **(G)** Western blot analysis for HO-1 in BV-2 cells after treatment with **5a** or GW501516 for 48 h. *P < 0.05, **P < 0.01 and ***P < 0.001 versus vehicle-treated control (one-way ANOVA with Dunnett's test). **(H-I)** Intracellular ROS levels assessed via DCFH-DA staining. BV-2 cells were treated with **5a** (5 μM) or GW501516 (5 μM) for 24 h followed by H_2_O_2_ (200 μM) for 20 min to induce oxidative stress.** (H)** Representative fluorescence images of DCFH-DA stained BV-2 cells. **(I)** Quantification of DCF fluorescence intensity in (H). ****P < 0.0001 versus H_2_O_2_-treated control (one-way ANOVA with Dunnett's test). Data are presented as mean ± SEM. GW: GW501516.

PPARδ regulates not only lipid metabolism but also inflammatory responses 14-18. Persistent microglial activation induces neuroinflammation, where excessive release of inflammatory factors causes neuronal cell death, contributing to the progression and severity of AD 28, 29. To investigate the effect of **5a** on the glial inflammatory responses, we treated BV-2 murine microglial cells with **5a** and GW501516. Compound **5a** did not affect the viability of BV-2 cells up to 20 μM and was less cytotoxic than GW501516 (Figure 3A). After pretreatment with **5a**, we induced inflammation by treating the cells with LPS and measured the levels of inflammatory mediators. LPS stimulation considerably increased the production of nitric oxide (NO) compared to that in the untreated control; this effect was suppressed by **5a** in a concentration-dependent manner (Figure 3B). Compound **5a** also reduced the LPS-induced increases in the mRNA and protein levels of inducible nitric oxide synthase (iNOS), a NO-synthesizing enzyme, and pro-inflammatory cytokines such as TNF-α and IL-6 (Figure 3C-E). We also evaluated the effect of **5a** on LPS-induced activation of the NF-κB p65 subunit that regulates the immune responses and production of inflammatory cytokines. Treatment of LPS increased the phosphorylation of p65, resulting in NF-кB activation. In contrast, **5a** prevented the increase in level of phosphorylated p65, maintaining its levels at a value similar to that in the LPS-untreated control (Figure 3F).

Besides direct inhibition of NF-кB, PPARδ indirectly inhibits the inflammatory responses by inducing the expression of antioxidant enzymes such as heme oxygenase-1 (HO-1) 44, 45. HO-1 is a major antioxidant enzyme and exhibits anti-inflammatory properties by suppressing the release of pro-inflammatory cytokines, reactive oxygen species (ROS), and activation of NF-кB p65 46, 47. Compound **5a** treatment increased the expression of HO-1 (Figure 3G) and consequently suppressed ROS production in BV-2 microglia (Figure 3H-I).

The anti-inflammatory effects of **5a** were also investigated in primary mouse astrocytes stimulated with LPS and interferon γ (IFNγ). Both **5a** and GW501516 exhibited no cytotoxicity in primary astrocytes up to 30 μM (Figure S3A). Similar to the findings in BV-2 microglia, treatment with **5a** significantly reduced NO release by inhibiting the expression of NO synthase and suppressed the production of pro-inflammatory cytokines, including TNF-α, IL-1β, and IL-6, in LPS/IFNγ-induced activated astrocytes (Figure S3B-E). These anti-inflammatory effects of **5a** on astrocytes were superior to those of GW501516 at the same concentration (Figure S3B-E). Taken together, these results indicate that **5a** potently downregulates inflammatory reactions in activated glial cells by reducing the levels of inflammatory mediators and NF-кB signaling and promoting antioxidant activity.

### 5a prevents locomotor and cognitive deficits in the LPS-induced neuroinflammation model

**Figure 4:**
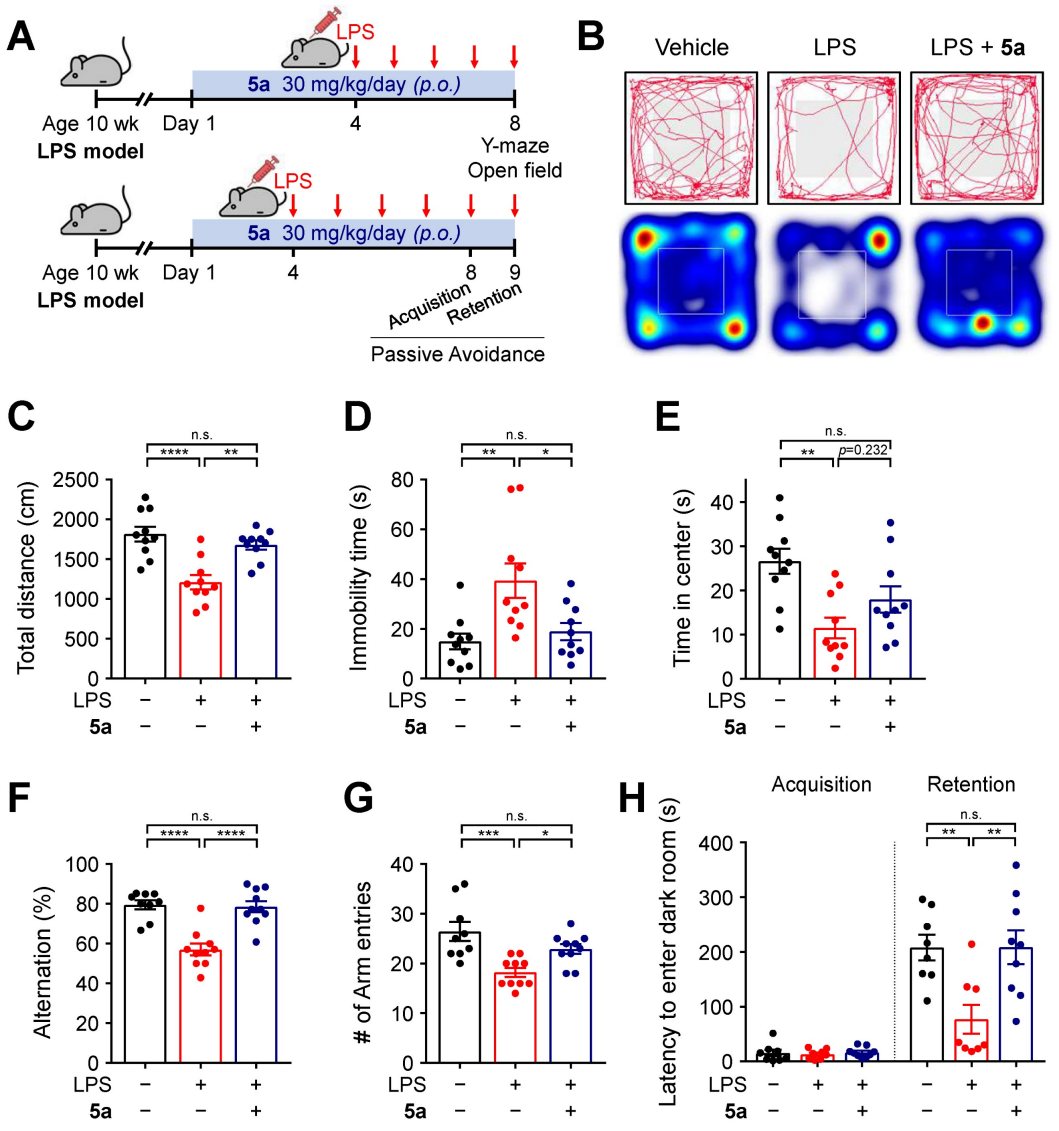
** 5a reverses behavioral deficits in LPS-induced neuroinflammation. (A)** Experimental protocol for behavioral tests after **5a** administration (30 mg/kg/day, *p.o.*) in mice with LPS-induced neuroinflammation (0.3 mg/kg/day, *i.p.*). **(B)** Representative track plots (top) and heat maps (bottom) in the open-field test. The center zone is indicated by gray squares in the track plot or white outline squares in the heat map. **(C-E)** Total traveled distance **(C)**, immobility time **(D)**, and time spent in the center **(E)** in the open-field test (*n* = 10 mice per group). **(F-G)** Percentage of spontaneous alternation **(F)** and number of arm entries **(G)** in the Y-maze test (*n* = 9-10 mice per group). **(H)** Step-through latency to enter a dark room in the passive avoidance test (*n* = 8-9 mice per group). *P < 0.05, **P < 0.01, ***P < 0.001 and ****P < 0.0001; n.s., not significant (one-way ANOVA with Tukey's test). Data are presented as mean ± SEM.

To further investigate the anti-inflammatory effects of **5a** *in vivo*, neuroinflammation was induced by daily intraperitoneal injections of LPS (0.3 mg/kg per day) in mice. The mice were previously treated with **5a** (30 mg/kg per day, *p.o.*) for 4 days (Figure 4A). Chronic administration of LPS causes sickness and depression-like behavior such as decreased motor activity and anxiety symptoms, leading to impaired cognitive function 48-50. We performed an open-field test to assess the exploratory behavior and locomotor activity of mice. LPS-treated mice showed a decrease in total traveled distance and an increase in immobility time when compared with vehicle-treated mice (Figure 4B-D). The administration of compound **5a** improved motor activity, which reached a level similar to that in vehicle-treated control mice (Figure 4B-D). In addition, **5a**-administered mice spent more time in the center of the open field than LPS-treated mice, suggesting that LPS-induced anxiety was alleviated (Figure 4B,E).

We next examined the cognitive ability of mice by performing a Y-maze test and a passive avoidance test. In the Y-maze test, mice treated with **5a** showed a higher percentage of spontaneous alternations reflecting better spatial learning and memory than LPS-treated mice (Figure 4F). **5a** administration increased the number of arm entries, which was reduced in LPS-treated mice, consistent with the improvement in motor activity identified in the open-field test (Figure 4G). The passive avoidance test was conducted for assessing hippocampus-dependent contextual learning and memory performance. When compared with the vehicle-treated control, LPS-treated mice showed a reduced step-through latency to enter the dark room owing to impaired memory (Figure 4H). However, administration of compound **5a** significantly prevented this decrease (Figure 4H), indicating that **5a** effectively restores cognitive deficits induced by LPS administration. Collectively, these results suggest that **5a** counteracts inflammation-induced motor deficits and cognitive impairments *in vivo*.

### Compound 5a ameliorates LPS-induced inflammation and glial activation *in vivo*

**Figure 5:**
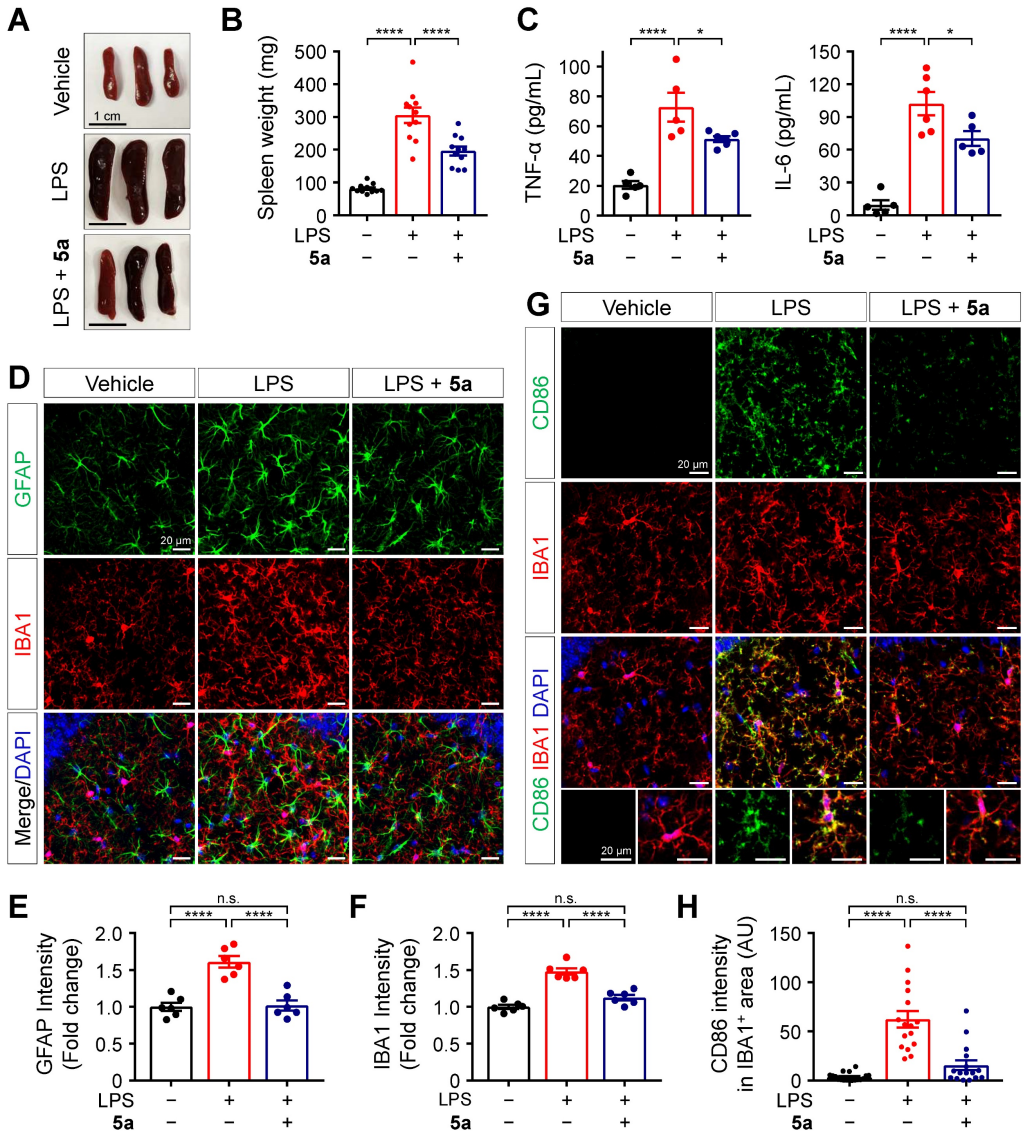
** 5a prevents LPS-induced inflammation and glial activation. (A-B)** Representative images **(A)** and mean weight **(B)** of dissected spleens from vehicle- and LPS-treated mice (0.3 mg/kg/day for 7 days, *i.p.*) with or without oral administrations of **5a** (30 mg/kg/day for 10 days) (*n* = 11 mice per group). **(C)** Plasma cytokine levels in vehicle- and LPS-treated mice with or without oral administrations of **5a** (*n* = 5-6 mice per group). *P < 0.05, and ****P < 0.0001 (one-way ANOVA with Dunnett's test). **(D)** Representative immunofluorescence images showing GFAP (green) and IBA1 (red) in CA1 region of hippocampus. **(E-F)** Quantification of immunoreactivity for GFAP **(E)** and IBA1 **(F)** in (D) (*n* = 6 brain sections from six mice per group). **(G)** Representative immunofluorescence images showing CD86 (green) and IBA1 (red) in the CA1 hippocampal region. **(H)** Mean intensity of CD86 in IBA1-positive areas (*n* = 16-18 brain sections from six mice per group). ****P < 0.0001; n.s., not significant (one-way ANOVA with Tukey's test). Data are presented as mean ± SEM.

Next, we evaluated the *in vivo* protective effects of **5a** against LPS-induced inflammation based on histological and biochemical analyses. C57BL/6N mice were intraperitoneally injected with LPS 0.3 mg/kg for 7 days, with or without oral administration of **5a** 30 mg/kg for 10 days. Following repeated LPS injections, the spleens were enlarged compared to those of vehicle-treated mice. Administration of **5a** reduced the size and weight of spleens in the LPS-treated mice (Figure 5A-B). Compound **5a** also prevented the LPS-induced increases in the plasma levels of pro-inflammatory cytokines TNF-α and IL-6 (Figure 5C).

Systemic inflammation triggers immune responses in the CNS 51. To determine whether **5a** treatment suppresses neuroinflammation and glial activation in the brain of mice, we immunostained the hippocampus and cortex regions with glial fibrillary acidic protein (GFAP) and ionized calcium-binding adaptor molecule 1 (IBA1), which are specific markers of astrocytes and microglia, respectively. Compared to the vehicle control, LPS increased the immunoreactivity for GFAP and IBA1 in both the hippocampus and cortex, and this effect was inhibited by **5a** (Figure 5D-F and Figure S4). We found that **5a** significantly reduced the expression of cluster of differentiation (CD)-86 (B7-2), a pro-inflammatory M1 marker, in IBA1-positive microglia compared with LPS-treated mice (Figure 5G-H). These results demonstrate that **5a** suppresses neuroinflammatory responses and glial activation induced by LPS.

### Compound 5a restores learning and memory impairment in AD mice

**Figure 6:**
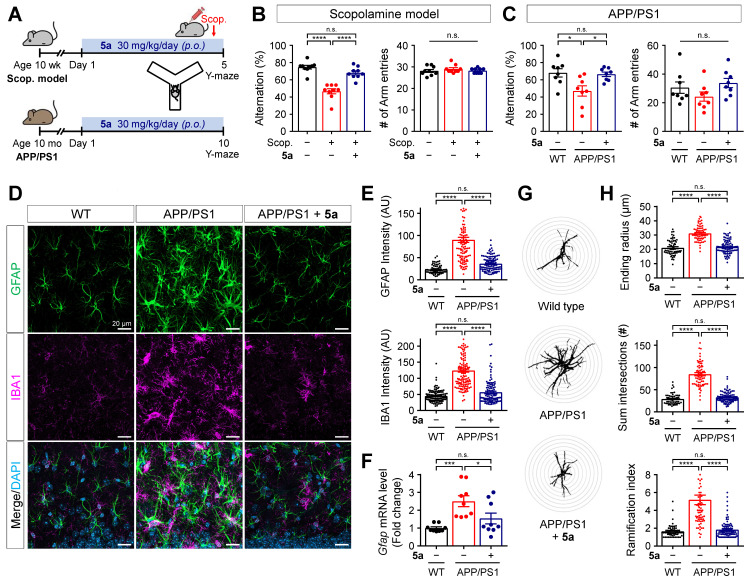
** 5a ameliorates cognitive impairment and AD-like features in mice with AD. (A)** Experimental protocol for the Y-maze test after oral administration of **5a** in mice with scopolamine-induced cognitive impairment (*n* = 9 mice per group) or APP/PS1 mice (*n* = 8 mice per group). **(B-C)** Percentage of spontaneous alternations (left) and number of arm entries (right) for mice with scopolamine-induced cognitive impairment **(B)** or APP/PS1 mice **(C)** treated with vehicle or **5a** in the Y-maze test. **(D)** Representative immunofluorescence images showing GFAP (green) and IBA1 (magenta) in the hippocampal dentate gyrus of WT and APP/PS1 mice treated with vehicle or **5a** (*n* = 8 brain sections from four mice per group). **(E)** Quantification of immunoreactivity for GFAP (top) and IBA1 (bottom) in (D). **(F)** Relative mRNA expression of *Gfap* in the hippocampus of WT and APP/PS1 mice treated with vehicle or **5a** (*n* = 9 mice per group). *Hprt* mRNA levels were used to normalize *Gfap* expression. **(G)** Representative images of Sholl analysis for GFAP-positive astrocytes in the hippocampal dentate gyrus of WT and APP/PS1 mice treated with vehicle or **5a** (*n* = 59-90 astrocytes in 8 brain sections from four mice per group). Interval of concentric circles, 3.125 μm. **(H)** Quantification of ending radius (top), sum of intersections (middle), and ramification index (bottom) of GFAP-positive astrocytes by Sholl analysis. *P < 0.05, ***P < 0.001 and ****P < 0.0001; n.s., not significant (one-way ANOVA with Tukey's test). Data are presented as mean ± SEM. Scop.: scopolamine; AU: arbitrary unit.

To examine if PPARδ agonist **5a** could alleviate AD-like pathology based on its anti-inflammatory properties, we investigated the therapeutic efficacy of **5a** using mice with scopolamine-induced cognitive impairment and 12-month-old APPswe/PSEN1dE9 (APP/PS1) double transgenic mice. Compound **5a** was orally administered in a dosage of 30 mg/kg per day for 5 days, prior to scopolamine injection, in scopolamine-treated mice and for 10 days in APP/PS1 mice. We performed the Y-maze or passive avoidance test to assess learning and memory performance during the **5a** treatment period (Figure 6A and Figure S5A). On day 4 before the scopolamine injection (1 mg/kg, *i.p.*), the Y-maze test revealed no differences in the percentage of alternations and total number of arm entries between the mouse groups, indicating that they had normal cognitive function and motility before scopolamine injection (Figure S5B). We observed spatial working memory deficits in scopolamine-treated and APP/PS1 mice, as reflected by the significantly reduced percentage of spontaneous alternations compared to the vehicle-treated and wild-type (WT) mice, respectively (Figure 6B-C). Treatment with** 5a** prevented the decrease in alternation percentage in both AD models without inducing any changes in the total number of arm entries (Figure 6B-C). In the passive avoidance test, the scopolamine-treated mice showed a lower step-through latency than vehicle-treated mice, indicating cognitive decline; **5a** administration mitigated this effect (Figure S5C).

We further investigated the cognitive improvement efficacy of **5a** and GW501516 when post-administered in a dose-dependent manner using the scopolamine-treated mice (Figure S6A). In the Y-maze (Figure S6B), passive avoidance (Figure S6C), and Morris water maze test (Figure S6D-I), **5a** significantly restored the memory impairment in scopolamine-treated mice at a dose of 10 mg/kg and also showed maximum efficacy at a dose of 30 mg/kg, improving cognitive ability to a level similar to that of vehicle-treated mice, thereby confirming the dose-dependent therapeutic effects of **5a**. In addition, **5a** exhibited superior *in vivo* efficacy in improving cognitive function compared to GW501516 when administered at the same dose of 30 mg/kg (Figure S6B-I). Taken together, administration of **5a** effectively restores learning and memory impairment in mice with scopolamine-induced cognitive impairment and APP/PS1 mice.

### Compound 5a mitigates reactive gliosis in APP/PS1 mice

Neuroinflammation accompanied by abnormal glial activation precedes neuropathological changes in AD and has been considered one of its pathogenic factors 29, 52. Both reactive astrocytes and activated microglia secrete pro-inflammatory cytokines, chemokines, and ROS, resulting in neurotoxicity 28, 53. Thus, we investigated whether treatment with **5a** interferes with neuroinflammation by regulating the activation of glial cells in AD mice. Compared with age-matched WT littermates, the APP/PS1 mice showed markedly increased immunoreactivity for GFAP and IBA1 in the molecular layer of the dentate gyrus (Figure 6D-E). The excessive glial activity was significantly inhibited by **5a** treatment (Figure 6D-E). APP/PS1 mice treated with **5a** also exhibited reduced levels of mRNA expression of *Gfap* in hippocampus homogenates, compared with the vehicle-treated APP/PS1 mice (Figure 6F). Additionally, Sholl analysis was performed to investigate the morphological characteristics of astrocytes and quantify the size and complexity of astrocytic processes, including maximum process lengths, sum of process intersections, and number of branch points. When compared with the astrocytes in WT mice, astrocytes in APP/PS1 mice were hypertrophied, as confirmed by a higher ending radius, sum of intersections, and ramification index (Figure 6G-H). In contrast, **5a** treatment significantly suppressed the astrocytic hypertrophy in APP/PS1 mice (Figure 6G-H). These results indicate that **5a** attenuates AD-like dysregulations including neuroinflammation and reactive gliosis in AD mice.

### 5a reduces amyloid burden by inhibiting beta-secretase 1 (BACE1) expression in APP/PS1 mice

**Figure 7:**
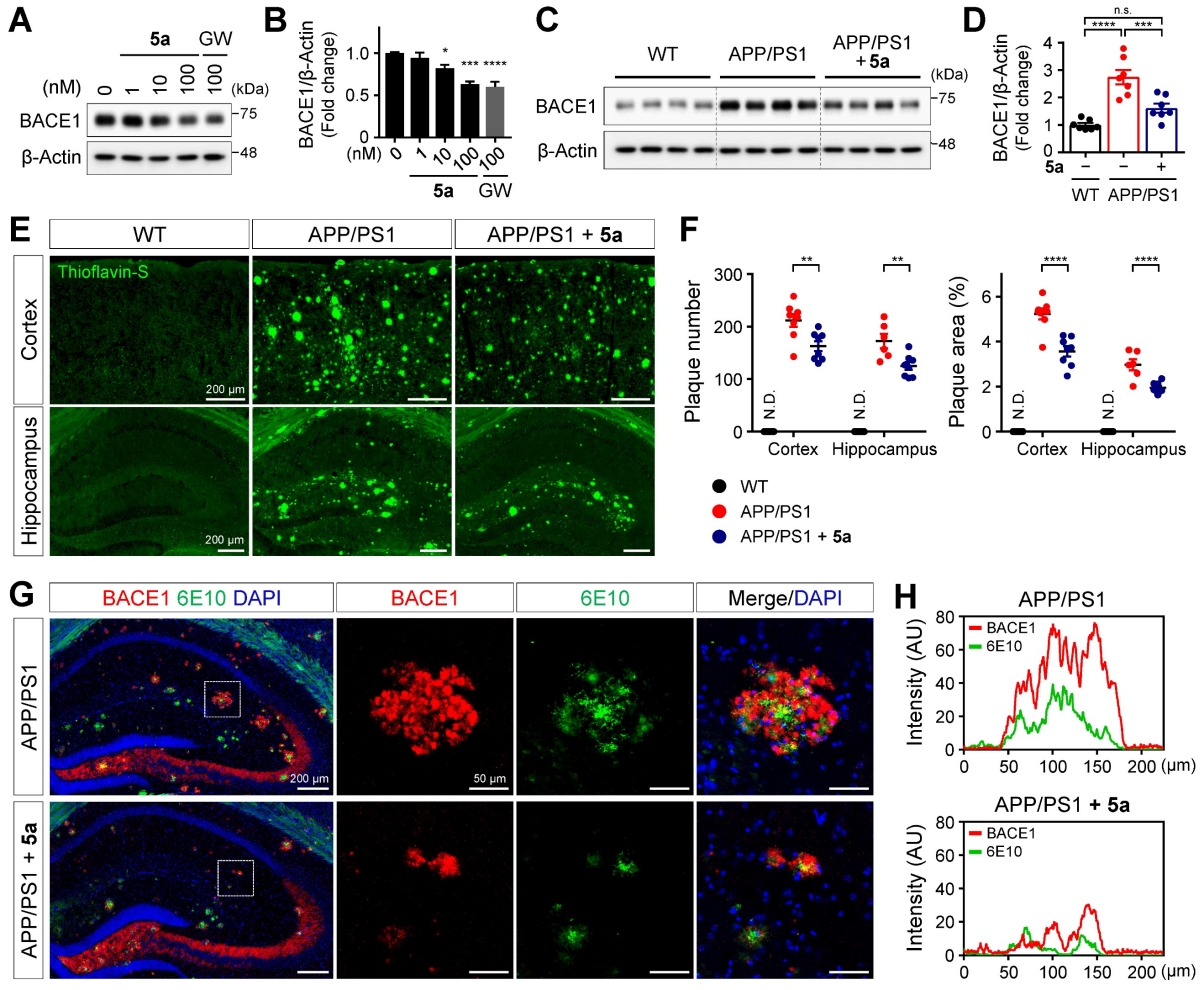
** 5a reduces BACE1 expression and amyloid burden in APP/PS1 mice. (A-B)** Western blot analysis for BACE1 in SH-SY5Y cells after treatment with **5a** or GW501516 for 24 h. *P < 0.05, ***P < 0.001 and ****P < 0.0001 versus vehicle-treated control (one-way ANOVA with Dunnett's test). **(C-D)** Western blot analysis for BACE1 in cortical homogenates of WT and APP/PS1 mice treated with vehicle or **5a** (*n* = 7 mice per group). **(E)** Thioflavin-S staining for Aβ plaques in the cortex and hippocampus of WT and APP/PS1 mice treated with vehicle or **5a** (*n* = 8 brain sections from four mice per group). **(F)** The number (left) and area (right) of thioflavin-S-stained Aβ plaques in the cortex and hippocampus. **P < 0.01, ***P < 0.001 and ****P < 0.0001; n.s., not significant (one-way ANOVA with Tukey's test). **(G)** Immunoreactivity of BACE1 (red) around 6E10 (green)-positive amyloid plaque in the hippocampus of APP/PS1 mice treated with vehicle or **5a** (*n* = 4 brain sections from four mice per group). Right, higher-magnification images of the dotted boxed area in the hippocampus. **(H)** Intensity profile plots showing colocalization of BACE1 with 6E10-positive amyloid plaques. Fluorescence intensity for BACE1 and 6E10 in APP/PS1 mice treated with vehicle (top) or **5a** (bottom) were measured using the magnified images in (G). Data are presented as mean ± SEM. N.D.: not detected; AU: arbitrary unit.

According to a previous study, PPARδ activation reduces the expression of BACE1 responsible for Aβ production in SH-SY5Y neuroblastoma cells 54. We also confirmed that **5a** dose-dependently reduced the expression of BACE1 in SH-SY5Y cells (Figure 7A-B). We next investigated whether **5a-**induced PPARδ activation inhibits beta-amyloid production in a mouse model of AD. Consistent with the results obtained following *in vitro* assessment, BACE1 levels were significantly diminished in cortical homogenates of **5a**-treated APP/PS1 mice compared to those in vehicle-treated APP/PS1 mice (Figure 7C-D). To examine whether **5a** could ameliorate the Aβ deposition in the brain, one of the major pathological features of AD, we performed thioflavin-S staining of brain tissues collected from WT and APP/PS1 mice treated with vehicle or **5a** and quantified the number and area of plaques in the cortex or hippocampus. Aβ plaques accumulated in the entire cortical and hippocampal region of 10-month-old APP/PS1 mice, whereas no Aβ plaques were detected in WT mice. (Figure 7E-F). Compared with vehicle-treated APP/PS1 mice, **5a**-treated APP/PS1 mice showed a significant reduction in the number and area of Aβ plaques in both the cortex and hippocampus, indicating that **5a** effectively reduced amyloid burden in APP/PS1 mice (Figure 7E-F).

In transgenic mouse models and patients with AD, BACE1-induced Aβ deposition subsequently causes BACE1 accumulation in neurons around amyloid plaques, leading to a positive-feedback loop in the amyloidogenic pathway 55-57. We observed that BACE1 was highly expressed around 6E10-positive amyloid plaques in association with overall Aβ accumulation in the hippocampus of APP/PS1 mice (Figure 7G-H). In contrast, **5a** administration significantly reduced the plaque-associated BACE1 accumulation in APP/PS1 mice (Figure 7G-H). Collectively, these results indicate that **5a** ameliorates amyloid-related pathology through BACE1 inhibition following PPARδ activation in APP/PS1 mice.

Additionally, we investigated whether PPARδ activation with **5a** could alleviate tau pathology, another pathological hallmark of AD. In mouse models of β-amyloidosis, although neurofibrillary tangles are not observed, hyperphosphorylation of tau occurs within dystrophic neurites surrounding amyloid plaques 58, 59. APP/PS1 mice treated with **5a** tend to decrease AT8-positive phosphorylated tau around thioflavin S-positive Aβ plaques, although this was insignificant (Figure S7A-B). However, **5a** treatment reduced the amount of amyloid plaques and their associated phosphorylated tau, thereby inhibiting overall tau hyperphosphorylation in the brain (Figure S7C-E).

## Discussion

In the present study, we designed a straightforward three-step approach to synthesize novel PPARδ-selective agonists with a selenazole heterocyclic core using commercially available 4-iodo-2-methylphenol. Compound **5a** was the most potent and selective PPARδ agonist with optimal *in vitro* ADMET and *in vivo* PK profiles. We determined the binding mode of **5a** to hPPARδ through an X-ray crystallographic analysis of the ligand-hPPARδ complex. Compound **5a** induced transcriptional activation of representative PPARδ target genes, including pyruvate dehydrogenase kinase 4 (*Pdk4*), angiopoietin-like 4 (*Angptl4*), and *Cd36*, confirming its effects on PPARδ activation *in vitro* and *in vivo* (Figure S8). We confirmed that **5a** inhibits excessive inflammatory responses in activated glial cells. Consistent with *in vitro* efficacy, **5a** treatment suppressed inflammatory responses and ameliorated behavioral defects in mice with LPS-induced neuroinflammation. The administration of **5a** also prevented glial hyperactivation and Aβ accumulation and subsequently restored learning and memory deficits in AD mice, suggesting its potential therapeutic applications for this pathology.

Although among other PPAR subtypes, PPARδ is the most abundantly expressed in the brain 8-10, its functional role in the CNS has been rarely studied, compared to PPARα or PPARγ. The role of PPARδ in inflammation and neurodegeneration is not fully understood owing to a lack of PPARδ-specific agonists 15. In our functional activity assay, **5a** showed a highly potent and specific agonistic effect on PPARδ with over 14,000-fold selectivity over PPARα and PPARγ. This PPARδ-selective agonist could be used for in-depth exploration to clarify PPARδ actions in the CNS.

Despite numerous efforts over decades, the development of effective treatments for AD remains a challenge. The AD drugs approved by the FDA so far have a limited efficacy, offering only symptomatic relief along with several side effects, and demonstrate short-term or questionable efficacy for cognitive improvement; similar concerns are observed with the currently approved amyloid-targeting antibodies 27, 60. AD is a complex degenerative disease that progresses over a long period of time and is caused by the interaction of multiple pathological pathways 28, 29. Therefore, overall targeting of these AD-related mechanisms, including neuroinflammation, nitrosative or oxidative stress, and reactive gliosis, could be an attractive therapeutic strategy in AD. In this study, we developed a novel PPARδ agonist **5a** and found that it is a potent anti-inflammatory agent that suppresses the expression of pro-inflammatory cytokines, NF-κB activation, and glial reactivity *in vitro* and *in vivo*. Compound **5a** reduces nitrosative stress by inhibition of iNOS and functions as an antioxidant, decreasing ROS accumulation owing to the induction of the antioxidant enzyme HO-1. These results support the beneficial effects of **5a** on multiple pathogenic pathways in AD.

PPARs regulate inflammation and immunity primarily through transrepression of other transcriptional factors such as NF-κB, signal transducers and activators of transcription (STATs), and activator protein 1 (AP1). Thus, their activation functionally antagonizes downstream signaling cascades 15. PPARδ regulates inflammatory responses by inhibiting NF-κB 61, 62, JAK2/STAT1 54, 63, and STAT3 signaling 62, resulting in reduced levels of iNOS, pro-inflammatory cytokines, chemokines, and vascular cell adhesion molecule 1 (VCAM1). PPARδ-deficient mice showed cognitive impairment associated with increased cytokine levels, NF-κB activation, and astrogliosis 32. These findings indicate that PPARδ has a crucial role in inflammation control, and a correlation exists between severe neuroinflammation and cognitive deficits. PPARδ activation with agonists such as GW0742 or GW501516 inhibits cytokine expression, ROS production, and inflammasome activation *in vitro*, in LPS-stimulated adipocytes 61 and brain cells 63, 64, and *in vivo*, as observed in the lung 65, heart 62, and liver 66 tissues of LPS-treated mice. In this study, we provide the first evidence for protective effects of **5a**-mediated PPARδ activation against cognitive decline accompanied by neuroinflammation. These effects include the amelioration of locomotor impairments, cognitive deficits, and glial reactivity induced by chronic LPS administration in mice.

Recent findings indicate that PPARδ is involved in the pathogenesis of AD 16-18. PPARδ agonists have previously shown therapeutic efficacy against AD-like pathologies in Aβ_1-42_-infused 31 or 5XFAD transgenic mouse models of AD 34, primarily through their anti-inflammatory effects. Interestingly, PPARδ deficiency was reported to accelerate tau hyperphosphorylation and amyloidogenesis, two pathological hallmarks of AD 32. PPARδ-null mice showed increased levels of BACE1, which is responsible for Aβ production, in the cortex 32. The peroxisome proliferator response element (PPRE), the binding site of all PPARs after activation, is present in the BACE1 promoter and PPARγ represses BACE1 transcription by direct binding to this PPRE 67. Ligand-activated PPARδ binds to the PPRE in the promoter region of suppressor of cytokine signaling 1 (SOCS1) rather than that of BACE1, thereby suppressing BACE1 expression through SOCS1-mediated inhibition of STAT1 signaling in SH-SY5Y cells 54. Herein, we report for the first time that PPARδ activation following administration of **5a** reduces BACE1 expression in the brain of APP/PS1 mice of AD. Further studies are still required to elucidate the precise mechanisms by which PPARδ regulation is involved in BACE1 transcription during APP processing in AD.

Hyperactivated microglia release inflammatory factors, causing neuroinflammation and worsening the progression of AD. However, before turning into a detrimental state under inflammatory and pathological conditions, microglia play a neuroprotective role by Aβ phagocytosis in the initial stage of AD 68, 69. PPARδ has been reported to be involved in apoptotic cell clearance by inducing opsonin genes in macrophages 70. In the 5xFAD mouse model of AD, pharmacological activation of PPARδ increases microglial association around Aβ plaques 34. We also observed a significant increase in the number of microglia per plaque in **5a**-treated APP/PS1 mice, indicating enhanced microglial recruitment to Aβ plaques (Figure S9A-B). In addition, **5a** treatment increased the phagolysosome area within plaque-associated microglia (Figure S9A, C) and the internalized Aβ area within the phagolysosome (Figure S9A, D), suggesting that activation of PPARδ with **5a** enhances microglial phagocytosis and contributes to Aβ clearance. Further studies are needed to investigate the underlying mechanisms of PPARδ-mediated regulation on microglial phagocytosis.

In conclusion, PPARδ activation with **5a** leads to favorable effects on amyloid burden via multiple mechanisms that inhibit excessive inflammation, oxidative stress, and increased levels of BACE1, which are all risk factors for Aβ deposition, thereby restoring cognitive impairment in the mouse models of AD. Our findings demonstrate that a selective PPARδ agonist **5a** could be investigated as a drug candidate for AD treatment.

## Materials and Methods

### General synthetic method

All reactions were performed in oven- and flame-dried glassware under a nitrogen (N_2_) atmosphere. Air- and moisture-sensitive reagents and solvents were transferred via syringes or cannulae into the reaction vessel through a rubber septum. Chemicals were obtained from commercial sources and used without further purification. Flash column chromatography was carried out using silica gel (particle size 230-400 mesh). Analytical thin-layer chromatography (TLC) was performed using silica gel 60 F254. TLC plates were visualized with UV light, by staining with 5% ammonium dimolybdate or panisaldehyde in ethanol and applying heat. ^1^H nuclear magnetic resonance (NMR, 300, 400, and 600 MHz) and ^13^C NMR (75 and 150 MHz) spectra were recorded for solutions in CDCl_3_ and MeOH-*d4*. Chemical shifts (*δ*) are expressed in parts per million (ppm) downfield from the internal reference, tetramethylsilane, or from residual CHCl_3_ in CDCl_3_. The purity of compounds was analyzed using high-performance liquid chromatography/mass spectrometry (HPLC/MS) spectra; mass spectrometry was conducted in positive electrospray ionization (ESI) mode on an LCMS-2020 system (Shimadzu). Column chromatography was performed using a CombiFlash^®^ Rf system with Re-diSep^®^ Rf (Teledyne ISCO). For the final compounds, further purification was performed by preparative HPLC on Kinetex^®^ 5 μm Biphenyl 100 Å (GX-281 HPLC system, Gilson; column tube: 250 mm × 21.2 mm ID) using ACN/H_2_O as eluent. The purity of target compounds was determined to be > 95% by analytical HPLC using a dual different wavelength UV detector. Starting materials were obtained from Sigma-Aldrich (St. Louis, MO, USA), or Alfa Aesar (Ward Hill, MA, USA). Solvents were obtained from Fisher Scientific (Hampton, NH, USA) or Sigma-Aldrich and were used without further purification unless noted otherwise.

### {2-Methyl-4-[4-methyl-2-(4-trifluoromethyl-phenyl)-selenazol-5-yl-methylsulfanyl]-phenoxy}-acetic acid (5a)

LiOH (0.6 mL solution 2 M [1.2 mmol]) was slowly added to a stirred solution of **4a** (528 mg, 1.0 mmol) in 15 mL tetrahydrofuran (THF) and 10 mL H_2_O at 0 °C. The reaction mixture was stirred at 0 °C until TLC indicated the completion of the reaction (approximately 2 h). The mixture was then diluted with water (10 mL), acidified with 0.5 M NaHSO_4_ (2.5 mL), and extracted with a mixed solvent containing EtOAc and THF (3:1, 15 mL). The organic layers were combined, dried (on Na_2_SO_4_), and concentrated. The residue was purified by column chromatography on silica gel with CH_2_Cl_2_/MeOH (10:1) and **5a** was obtained as a white solid (460 mg, 92%). ^1^H NMR (600 MHz, MeOH-*d4*) *δ:* 7.95 (d, 2 H, *J* = 8.2 Hz), 7.70 (d, 2 H, *J* = 8.3 Hz), 7.19-7.17 (m, 2 H), 6.71 (d, 1 H, *J* = 8.4 Hz), 4.55 (s, 2 H), 4.22 (s, 2 H), 2.20 (s, 3 H), 2.15 (s, 3 H). ^13^C NMR (150 MHz, MeOH-*d4*) *δ:* 171.4, 158.4, 152.4, 151.6, 140.9, 140.8, 137.0, 133.4, 129.3, 128.1, 127.2, 125.8, 112.9, 67.4, 35.2, 16.5, 15.4. LC/MS (ESI^+^) calculated for C_21_H_18_F_3_NO_3_SSe identified as [M + H]^+^ ion as *m*/*z* 500.39. Found: *m*/*z* 502.03 (isotope).

### {2-Methyl-4-[4-methyl-2-(4-trifluoromethyl-phenyl)-selenazol-5-yl-methylselanyl]-phenoxy}-acetic acid (5b)

LiOH (0.6 mL solution of concentration 2 M [1.2 mmol]) was slowly added to a stirred solution of 4b (575 mg, 1.0 mmol) in 15 mL THF and 10 mL H_2_O at 0 °C. The reaction mixture was stirred at 0 °C until TLC indicated the completion of the reaction (approximately 2 h). The mixture was then diluted with water (10 mL), acidified with 0.5 M NaHSO_4_ (2.5 mL), and extracted with a mixed solvent containing EtOAc and THF (3:1, 15 mL). The organic layers were combined, dried (on Na_2_SO_4_), and concentrated. The residue was purified by column chromatography on silica gel with CH_2_Cl_2_/MeOH (10:1) and **5b** was obtained as a white solid (492 mg, 90%). ^1^H NMR (600 MHz, CDCl_3_) *δ*: 7.87 (d, 2 H, *J* = 8.1 Hz), 7.62 (d, 2 H, *J* = 8.2 Hz), 7.30 (s, 1 H), 7.22 (d, 1 H, *J* = 8.3 Hz), 6.55 (d, 1 H, *J* = 8.4 Hz), 4.59 (s, 2 H), 4.17 (s, 2 H), 2.20 (s, 3 H), 2.08 (s, 3 H). ^13^C NMR (150 MHz, CDCl_3_) *δ*: 173.4, 169.6, 156.5, 151.4, 139.2, 138.4, 133.4, 128.6, 127.1, 126.2, 123.2, 120.2, 112.0, 65.5, 25.6, 16.2, 15.3. LC/MS (ESI^+^) calculated for C_21_H_18_F_3_NO_3_Se_2_ identified as [M + H] ^+^ at *m*/*z* 547.29. Found: *m*/*z* 550.00 (isotope).

### Protein production and crystallization

A human recombinant DNA PPARδ LBD (residues 171 - 441) was designed. Following codon optimization, it was inserted into a pET28a vector. N-terminal 6 His-tagged Human PPARδ LBD (171-441) was overexpressed in *Escherichia coli* BL21-Star (DE3) cells. The cells were grown at 37 °C to an optical density at 600 nm (OD_600_) of 1.0 in Terrific Broth (TB) medium containing 50 μg/mL kanamycin and induced with 0.5 mM isopropyl-β-D-thiogalactopyranoside (IPTG). Inclusion bodies formed by the overexpressed proteins were studied 16 h after IPTG induction at 18 °C, followed by harvesting using centrifugation at 6000 rpm and 4 °C for 15 min. The cell pellets were resuspended in lysis buffer (50 mM Bis-Tris propane pH 8.5, 500 mM NaCl, 10% glycerol, and 5 mM β-mercaptoethanol) and lysed in an ultrasonic processor. The cell lysate was centrifuged at 15,000 rpm for 40 min at 4 °C. N-terminal His-tagged Human PPARδ LBD in the supernatant fraction was purified via nickel affinity chromatography followed by gel filtration. The protein was concentrated to 8 mg/mL in 20 mM HEPES (final pH 7.5), 500 mM ammonium acetate, and 10 mM β-mercaptoethanol for crystallization. Crystallization was carried out using the hanging-drop vapor diffusion method. Apo crystals of human PPARδ LBD were obtained from a 4 μL drop consisting of 2 μL well solution [5 - 15% (w/v) PEG 8000, 40 mM Bis-tris propane pH 7.0 - 9.5, 2.5% 1,2-propanediol, 200 mM KCl, and 10 mM dithiothreitol (DTT)] and 2 μL of protein, with 10% n-Hexyl-β-D-glucopyranoside 1 M. The crystals appeared within 2-3 days. The co-crystal complex-**5a** was obtained by soaking the apo crystal in 250 μM selenazole for 24 h in the presence of 10% PEG 8000, 40 mM Bis-tris propane (pH 8.5), 2.5% 1,2-propanediol, 200 mM KCl, and 10 mM DTT at 18 °C. For the diffraction test, crystals were cryo-protected by dipping into a solution of 10% PEG 8000, 40 mM Bis-tris-propane (pH 8.5), 2.5% 1, 2-propanediol, 200 mM KCl, 10 mM DTT, and 20% glycerol (v/v).

### Data collection, structure determination, and refinement

Diffraction data were collected at 1.70 Å resolution, using in-vacuum undulator beamline BL-5C of the Pohang Accelerator Laboratory (PAL), South Korea. HKL2000 (HKL Research, Inc.) was used for integration and scaling. Molecular replacement was used to determine the structure of human PPARδ LBD bound with **5a** using a previously known human PPARδ LBD structure (PDB entry: 5U3R) without water and ligand, as a search model. Rigid body refinement, followed by simulated annealing at 5000 K, was conducted using PHENIX (Python-based Hierarchical ENvironment for Integrated Xtallography). Subsequently, refinement was conducted in alternating cycles of manual model building in COOT (Crystallographic Object-Oriented Toolkit), followed by restrained refinement in PHENIX until the R factors converged. Data collection and refinement statistics are shown in Table S5. The coordinate and structural factors of the complex structure have been deposited in the Protein Data Bank (PDB code: 5Y7X). Structure visualization was performed with Chimera 71.

### CYP inhibition assay

All incubations were performed in duplicate, and the mean values were used for analysis. Cocktail incubations were carried out using eight probe substrates of CYP1A2, CYP2C9, CYP2C19, CYP2D6, and CYP3A4: phenacetin, tolbutamide, mephenytoin, dextromethorphan and midazolam respectively, followed by tandem mass spectrometry. Briefly, an incubation reaction was performed using 0.25 mg/mL human liver microsomes in a final incubation volume of 100 μL. The incubation medium contained 100 mM phosphate buffer (pH 7.4) and probe substrates. The incubation mixture containing various inhibitors (10 μM) was pre-incubated for 5 min. After pre-incubation, an NADPH-regenerating system was added. After incubation at 37 °C for 15 min, the reaction was stopped by placing the incubation tubes on ice and adding 40 μL of ice-cold acetonitrile. The incubation mixtures were centrifuged at 10,000 *g* and 4 °C for 5 min. Aliquots of the supernatant were injected onto an LC-MS/MS system. The CYP-mediated activities in the presence of inhibitors were expressed as a percentage of the corresponding control value 72.

### Metabolic stability assay in liver microsomes

The metabolic stability assay was performed by incubation of human and selected animal liver microsomes (dog, rat, and mouse) with the test compound at a final concentration of 1 μM, in the presence of 0.5 mg/ml microsomal protein and NADPH-regeneration system, in a total volume of 100 μL phosphate buffer 100 mM (pH 7.4) at 37 °C. The incubation was started by introducing the NADPH-regeneration system and terminated by adding 40 μL of ice-cold acetonitrile at 0- and 30 min. Precipitated proteins were removed by centrifugation at 10,000 *g* and 4 °C for 5 min. Aliquots of the supernatant were injected onto an LC-MS/MS system. Incubations terminated prior to addition of the NADPH-regeneration system (time point, 0 min) were used as standards, defined as 100%. Percent of the parent compound remaining was calculated by comparing peak areas 73.

### hERG channel inhibition assay

An hERG channel binding assay was performed using the Predictor hERG Fluorescence Polarization Assay Kit (PV5365, Invitrogen). Briefly, for measuring IC_50_, compounds were serially diluted (16 points, 3-fold) and then mixed for 4 h at 25 °C with a reaction mixture containing hERG membrane, fluorescence tracer red dye, and fluorescence polarization buffer. Fluorescence intensity (excitation at 530 nm, emission at 590 nm) was measured using a multi-mode microplate reader Synergy Neo (Biotek). E-4031 was used as the reference positive standard (IC_50_ = 10 - 90 nM) 74.

### Pharmacokinetics studies

Sprague-Dawley rats (weighing 250-300 g, 7-week-old) were fasted for 16 h and used for experiments. Before administration of compound **5a**, blood was collected from the jugular vein and used as a blank control. For oral administration, four rats received the compound suspended in 10% DMSO, 15% water, and 75% PEG 400 at a dose of 10 mg/kg via oral gavage. For intravenous administration, a dose of 1 mg/kg of the compound was injected into the caudal vein. The volumes of administration were 600 μL for oral and 200 μL for intravenous administration. Blood from the jugular vein was collected into a heparinized tube at 0.08, 0.25, 0.5, 1, 2, 4, 6, and 8 h after compound administration. Plasma samples were prepared from the extracted blood by centrifugation at 12,000 rpm for 15 min. Then, 20 μL of plasma was mixed with 80 μL of acetonitrile containing internal standard and centrifuged at 14,000 rpm for 5 min. Collected plasma supernatants were loaded into triple quadrupole LC-MS/MS (Triple Quad 5500, Applied Biosystems) and the amount of contained compound was measured. The calibration curve had a range of 5 to 1000 ng/mL, and the lower limit of quantification was 5 ng/mL. Non-compartmental analysis was used to determine pharmacokinetic parameters (Phoenix WinNolin ver 6.4, Pharsight).

### Ames MPF mutagenicity assay

An Ames microplate format (MPF) 98/100 mutagenesis assay kit (Xenometrix) was used to test mutagenic activity. The TA98 / TA100 *Salmonella* strain was inoculated into the bacterial culture medium and cultured overnight. The *Salmonella* test strain was then exposed to six concentrations of the test compound (plus positive and negative controls). Samples were prepared in 96-well plates in sterile DMSO. The cultured bacteria, test compound, and S9 mixture were incubated for 90 min at 37 °C in a shaking incubator. The mixture was then treated with an indicator medium and dispensed into 384-well plates. The plates were incubated at 37 °C for 48 h. The number of wells containing revertant colonies was counted for each dose.

### Cell culture

For the *in vitro* co-transfection assay, CV-1 monkey kidney cells were cultured in Dulbecco's modified Eagle's medium (DMEM) supplemented with 10% resin-charcoal-stripped fetal bovine serum (FBS), 100 U/mL penicillin, and 100 g/mL streptomycin. To investigate the anti-inflammatory effects of **5a**, BV-2 microglial cells were cultured in RPMI 1640 (Biowest) supplemented with 10% heat-inactivated FBS (Biowest), 2.05 mM L-glutamine (Gibco), and 100 U/mL penicillin/streptomycin (Gibco). Primary mouse astrocytes were isolated from postnatal day 1 C57BL/6N mouse pups. The cerebral cortices collected with the meninges removed were triturated by gentle pipetting and centrifuged. Primary astrocytes were resuspended and cultured in DMEM supplemented with 10% heat-inactivated FBS (Biowest), 10% heat-inactivated horse serum (Gibco), and 100 U/mL penicillin/streptomycin (Gibco). On days *in vitro* (DIV) 4, floating other cell types were removed by tapping the culture dish, and astrocytes were used for experiments on DIV6. To investigate the effects of **5a** on BACE1 expression, SH-SY5Y human neuroblastoma cells (Korean Cell Line Bank, 22266) were cultured in minimum essential medium (MEM) supplemented with 10% heat-inactivated FBS (Biowest), 25 mM HEPES (Gibco), 25 mM sodium bicarbonate (Gibco) and 100 U/mL penicillin/streptomycin (Gibco). Cells were maintained at 37 °C in a humidified atmosphere containing 5% CO_2_.

### *In vitro* transfection assay

CV-1 cells were seeded at 6 × 10^3^ cells per well in 96-well culture plates and transfected once they reached 70% confluency. The cells were washed with serum-free medium and then transfected with a plasmid mixture containing human PPAR expression vector, β-galactosidase, and TK-PPRE-Luc vector by Superfecta reagent (QIAGEN). Twenty-four hours post-transfection, cells were washed with serum-free DMEM and incubated with freshly delipidated 5% FBS DMEM supplemented with either compounds or DMSO vehicle for 24 h. After incubation, cell lysates were obtained using cell lysis buffer, and a luciferase activity assay was performed upon substrate addition using a Microlumat Plus Luminometer (Berthold). The luciferase activity was normalized with β-galactosidase activity using an ONPG buffer. All assays were performed in triplicate.

### Cytotoxicity assay

The *in vitro* cytotoxicity of **5a** was evaluated using the EZ-Cytox assay kit (DoGenBio, South Korea). BV-2 microglia or primary mouse astrocytes were seeded in 96-well clear microplates and treated with **5a** or GW501516 for 24 h at 37 °C. Absorbance was measured at 450 nm, after 1 h of incubation with EZ-Cytox containing water-soluble tetrazolium salt (WST), using a SpectraMax^®^*i*3 microplate reader (Molecular Devices).

### Griess assay for nitrite measurement

The nitric oxide (NO) released into the culture medium was quantified by measuring nitrite levels using the Griess method. BV-2 microglia were treated with **5a** or GW501516 for 3 h and stimulated with LPS (0.2 μg/ml) for another additional 24 h. Primary mouse astrocytes were pretreated with compounds for 6 h and stimulated with LPS (100 ng/ml)/IFNγ (1 ng/ml) for 42 h. Culture media were collected and centrifugated at 2356 *g* for 3 min to remove large cell debris. The supernatants were incubated with equal amounts of sulfanilamide solution (1% sulfanilamide in 5% phosphoric acid) for 5 min in a 96-well microplate, followed by NED solution (0.1% N-1-napthylethylenediamine dihydrochloride in water) for 5 min at room temperature (20-25 °C) in the dark. Nitrite concentrations were determined by measuring the absorbance at 540 nm using a SpectraMax^®^*i*3 microplate reader (Molecular Devices) based on a standard reference curve prepared with 0.1 M sodium nitrite.

### Quantitative real-time reverse-transcription PCR (qRT-PCR) analysis

Total RNA was isolated using TRIzol^®^ (Invitrogen). Genomic DNA was removed and complementary DNA was synthesized from 500 ng total RNA using the iScript^™^ gDNA Clear cDNA Synthesis Kit (Bio-Rad). Real-time PCR was performed using the CFX Connect^™^ Real-Time PCR Detection System (Bio-Rad) using iQ^™^ SYBR® Green Supermix (Bio-Rad). The hypoxanthine phosphoribosyltransferase (*Hprt*) was used to normalize mRNA expression levels. Primer sequences used are described in Table S6.

### Western blotting

Total proteins were extracted from cells or mouse brain tissues using radioimmunoprecipitation assay (RIPA) buffer (Sigma-Aldrich) containing a protease and phosphatase inhibitor cocktail (Roche Diagnostics). After 30 min of incubation with RIPA buffer on ice, lysates were centrifuged at 15800 *g* and 4 °C for 20 min and supernatants were collected. Protein concentrations were determined using Pierce^®^ BCA Protein Assay Kit (Thermo Fisher Scientific). Protein samples (10 μg) were separated on 10% SDS-PAGE gels and transferred to polyvinylidene fluoride (PVDF) membranes (Millipore). The membranes were blocked in 5% skim milk (Millipore) in TBST (10 mM Tris-HCl, pH 7.5, 150 mM NaCl, and 0.1% Tween^®^ 20) for 1 h at room temperature and incubated overnight at 4 °C with primary antibodies. After washing three times with TBST, the membranes were incubated with horseradish peroxidase-conjugated anti-mouse or anti-rabbit IgG (GeneTex) for 1 h at room temperature and then developed with SuperLumia ECL HRP Substrate solution (Abbkine) using the Amersham Imager 600 (GE Healthcare). The western blot bands were analyzed using ImageJ software (NIH) and β-Actin or glyceraldehyde 3-phosphate dehydrogenase (GAPDH) were used as a loading control. Primary antibodies used are described in Table S6.

### Enzyme-linked immunosorbent assay (ELISA)

The concentration of pro-inflammatory cytokines in the cell culture medium or mouse plasma was determined using ELISA kits for mouse TNFα (Invitrogen, 88-7324-88) and IL-6 (Invitrogen, 88-7064-88). BV-2 microglia were treated with **5a** or GW501516 for 9 h followed by LPS (0.2 μg/ml) stimulation for 18 h. Primary mouse astrocytes were treated with compounds for 24 h followed by LPS (10 ng/ml)/IFNγ (1 ng/ml) stimulation for 12 h, and the culture medium was used for analysis. Mouse plasma samples were obtained by centrifuging blood collected from the heart at 848 *g* and 4 °C for 15 min. Absorbance was measured at 450 nm using a SpectraMax^®^*i*3 microplate reader (Molecular Devices).

### Fluorescence imaging of intracellular ROS

Intracellular ROS accumulation was monitored using the cell-permeable fluorescent probe DCFH-DA (Sigma-Aldrich). BV-2 microglia were seeded in 96-well black plates and treated with **5a** or GW501516 for 24 h at 37 °C. The cells were loaded with 40 μM DCFH-DA in culture medium for 40 min at 37 °C in the dark. Then, 200 μM H_2_O_2_ was added to the medium for an additional 20 min at 37 °C. After washing with phosphate-buffered saline (PBS), cells were imaged with an ImageXpress Pico (Molecular Devices) and fluorescence intensity was quantified using the ImageJ software (NIH).

### Animals and treatment

All mice and rats were housed in a temperature- and humidity-controlled environment with a 12 h light/dark cycle and ad libitum access to food and water. All procedures were performed in accordance with the National Institutes of Health Guide for the Care and Use of Laboratory Animals and approved by the Institutional Animal Care and Use Committee of Korea Institute of Science and Technology (KIST-IACUC-2022-020-1). For the pharmacokinetics study, Sprague-Dawley rats were purchased from Koatech (South Korea) and maintained in a specific pathogen-free facility (Laboratory Animal Center, Daegu-Gyeongbuk Medical Innovation Foundation). For the LPS-induced neuroinflammation model or scopolamine-induced AD model, male C57BL/6N mice were obtained from DBL (South Korea). APP/PS1 (APP_swe_/PSEN1dE9) transgenic mice from the Jackson Laboratory (USA; stock number 004462) were maintained as hemizygotes on the C57BL/6; C3H genetic background at the Research Animal Resources Center of KIST. 10-week-old C57BL/6N and 12-month-old APP/PS1 were used in behavioral experiments with sex- and age-matched controls.

Compound **5a** and GW501516 (Combi-Blocks, QJ-9419) were dissolved in distilled water and orally administered once per day for the duration of the experiment. For the neuroinflammation model, C57BL/6N mice were intraperitoneally injected daily with 0.3 mg/kg LPS (Sigma-Aldrich, L2880) dissolved in saline (0.9% sodium chloride), for 6 consecutive days. In a non-genetic AD model, we induced acute memory deficits in C57BL/6N mice by intraperitoneal injection with 1 mg/kg of scopolamine hydrobromide (Sigma-Aldrich, S0929) dissolved in saline, 30 min before the behavioral tests. At the end of the experimental period, mice were deeply anesthetized with 2% avertin (500 mg/kg, *i.p.*) and transcardially perfused with saline. Brains were collected from each mouse, and the hippocampus and cortex were isolated and stored at -80 °C for biochemical analysis. Brain hemispheres were postfixed in 4% paraformaldehyde overnight at 4 °C, then placed in 30% sucrose solution for 48 h at 4 °C for cryoprotection and used for histological analysis.

### Open-field test

The locomotor activity in mice was assessed using the open-field test. Mice were placed in the center of a white square chamber (40 cm × 40 cm × 30 cm). All movements were recorded for 5 min and analyzed by an automated tracking software Ethovision XT 11.5 (Noldus). The center zone was defined as a 20 cm × 20 cm area in the center of the chamber.

### Y-maze test

The Y-maze test was used to evaluate the spatial learning and memory performance of mice. The Y-maze apparatus consisted of three identical arms disposed at a 120° angle from each other (41 cm long and 7 cm wide with 15 cm high walls). Mice were placed at the end of one arm and allowed to move freely in the maze for 10 min. The arm entries were manually recorded. Spontaneous alternation was defined as consecutive entries into three different arms and the percentage of alternation was calculated based on the following equation: alternation (%) = number of alternations/(total arm entries - 2) × 100.

### Passive avoidance test

The passive avoidance test was performed in two compartments, a light room and a dark room, separated by a guillotine door (GEMINI Avoidance System, San Diego Instruments). On the first experimental day, mice were placed in the light room for the acquisition trial. After exploration for 30 s, the guillotine door was raised and the mice were allowed to enter the dark room spontaneously. When the mice crossed the door, the door was closed and 3 s later an electric foot shock (0.4 mA, 1 s duration) was applied. The mice remained in the dark room for an additional 30 s and then were returned to their home cages. Twenty-four hours after the acquisition trial, the mice were placed again in the light room and the door was raised after 15 s. The step-through latency to enter the dark room was automatically measured using GEMINI software (San Diego Instruments).

### Morris water maze test

The spatial learning and memory performance of mice was assessed using the Morris water maze test. A white circular tank (150 cm in diameter, 58 cm in height) was filled with water at a temperature of 24 - 25 °C and a white circular platform (20 cm in diameter) was placed 1.5 cm lower than the water surface, and white non-toxic tempera paint was dissolved in water so that the mice could not visually determine the location of the platform. The water maze was divided into quadrants, and mice were released at each of the four points. To enable the mice to recognize each direction, spatial cues of different shapes were placed on the wall in the north, east, and south directions. In acquisition tests for 7 days, mice were trained to find the platform 4 times a day at each releasing point for 60 s. In a probe test on day 8, mice were allowed to swim freely for 100 s with the platform removed. All trials were recorded and automatically analyzed using the EthoVision XT 11.5 (Noldus). The target zone in the probe test was defined as an area with a radius of 30 cm centered on the platform during the acquisition test.

### Immunofluorescence staining

Fixed mouse brains were embedded in an optimal cutting temperature compound (Leica, 3801480) and serially sectioned at 30 μm in a cryostat (Thermo Fisher Scientific, HM525 NX). Coronal hippocampal sections were stored in a storage solution (30% glycerol, 30% ethylene glycol, and 30% 0.2 M sodium phosphate buffer, pH 7.2) at 4 °C until use. After washing three times with PBS, the sections were blocked for 1 h with blocking buffer (0.3% Triton X-100 and 4% normal donkey serum in 0.1 M PBS) at room temperature, followed by incubation with primary antibodies in blocking buffer overnight at 4 °C. After washing with PBS, the sections were incubated with corresponding fluorescent secondary antibodies (Jackson ImmunoResearch, Alexa Fluor) for 1 h at room temperature and washed three times with PBS. Nuclei were counterstained with 4′,6-diamidino-2-phenylindole (DAPI; Invitrogen) during the second washing step. Sections were mounted with fluorescent mounting medium (Dako). Fluorescent images were acquired with the ImageXpress Pico (Molecular Devices; 20× magnification) or LSM 800 confocal microscope (Carl Zeiss; 40× magnification). Primary antibodies used are described in Table S6.

### Image quantification

Z-stack projected images (20 μm thickness with a step size of 2 μm or 4 μm) were analyzed using the ImageJ software (NIH). To quantify glial reactivity, the mean fluorescence intensity of GFAP or IBA1 was measured. To evaluate microglial M1 polarization, the mean fluorescence intensity of CD86 was measured in the IBA1 staining-positive area. In CD86 and IBA1 double-stained sections, IBA1-immunostained pixels were defined as a region of interest after thresholding. For the analysis of plaque-associated BACE1, the fluorescence intensity of BACE1 or 6E10 was measured along a 200 μm^2^ area around 6E10-positive amyloid plaque using the 'Plot Profile' function of ImageJ software (NIH). To quantify tau phosphorylation levels, the AT8-stained area was measured within a 50 μm range of the thioflavin S-positive plaque. To assess microglial Aβ phagocytosis, within a 50 μm range of the 6E10-positive plaque, the number of IBA1-positive microglia was counted and the CD68-positive area was measured in the IBA1 or 6E10-positive area.

### Morphological analysis of astrocytes

Confocal z-stack images (20 μm thickness with 2 μm step size; under 40× magnification) were analyzed using the 'Sholl Analysis' function of ImageJ software (NIH). Individual GFAP-positive astrocytes in the hippocampal dentate gyrus were maximally projected and converted to binary images by thresholding. Continuous concentric circles were drawn at 3.125 μm intervals from the center of the soma. The maximum length of astrocytic processes (ending radius), total number of intersections per concentric circle (sum intersections), and ramification index were automatically calculated.

### Thioflavin-S staining

Aβ plaques in APP/PS1 mouse brains were labeled with thioflavin-S (Sigma-Aldrich, T1892). Coronal brain sections were incubated for 8 min with 1 mM thioflavin-S dissolved in 50% ethanol and washed two times with 80% ethanol and three times with PBS. Stained sections were mounted on slides with fluorescent mounting medium (Dako) and visualized with the ImageXpress Pico (Molecular Devices). For plaque analysis, the number and area of plaques in the cortex or hippocampus were quantified using the 'Analyze Particles' function of ImageJ software (NIH).

### Statistical analysis

Data were analyzed with GraphPad Prism 7 software and presented as mean ± SEM. The statistical significance of differences between the two groups was determined using an unpaired two-tailed Student's t-test. Multiple comparisons between groups were performed with one-way analysis of variance (ANOVA) followed by Tukey's or Dunnett's *post-hoc* test and repeated measures one-way ANOVA followed by Fisher's LSD test. The statistical significance level is displayed as asterisks (*P < 0.05, **P < 0.01, ***P < 0.001, and ****P < 0.0001; n.s. not significant).

## Supplementary Material

Supplementary methods, results, spectra, figures.

## Figures and Tables

**Table 1:** Potency and selectivity of GW501516, 5a, and 5b for human PPARs.

Compounds	Potency (EC_50_ , nM)			Selectivity index^*^	
	hPPARα	hPPARδ	hPPARγ	hPPARα/hPPARδ	hPPARγ/hPPARδ
**GW501516**	300	1.2	1200	250	1000
**5a**	>10000	0.7	>10000	>14000	>14000
**5b**	>10000	4.3	>10000	>2300	>2300

^*^ Selectivity index represents the selectivity for the PPARδ isoform and is given as the ratio of EC_50_(hPPARα)/EC_50_(hPPARδ) or EC_50_(hPPARγ)/EC_50_(hPPARδ). EC_50_: half maximal effective concentration; nd: not determined.

## Abbreviations

AD: Alzheimer's disease
ADMET: absorption, distribution, metabolism, excretion, and toxicity
ANGPTL4: Angiopoietin-like 4
Aβ: amyloid β
BACE1: beta-secretase 1
CD: cluster of differentiation
CNS: central nervous system
CYP: cytochrome P450
DTT: dithiothreitol
EC_50_: half maximal effective concentration
FBS: fetal bovine serum
GFAP: glial fibrillary acidic protein
hERG: human ether-à-go-go-related gene
HO-1: heme oxygenase-1
HPLC: high-performance liquid chromatography
IBA1: ionized calcium-binding adaptor molecule 1
IFNγ: interferon γ
IL: interleukin
iNOS: inducible nitric oxide synthase
LBD: ligand-binding domain
LPS: lipopolysaccharide
MS: mass spectrometry
NF-κB: nuclear factor-κB
NO: nitric oxide
PDK4: pyruvate dehydrogenase kinase 4
PK: pharmacokinetic
PPAR: peroxisome proliferator-activated receptor
PPRE: peroxisome proliferator response element
ROS: reactive oxygen species
THF: tetrahydrofuran
TNF: tumor necrosis factor
WT: wild type
