# Supplementary material for: Highly potent and selective PPARδ agonist reverses memory deficits in mouse models of Alzheimer's disease
Source: Theranostics. 2024 Sep 16;14(16):6088–108. doi: 10.7150/thno.96707 (PMC11488110; doi:10.7150/thno.96707)
Supplement: Supplementary file 1 — Supplementary methods, results, spectra, figures. [file thnov14p6088s1.pdf]

Supplementary Information

Highly potent and selective PPARδ agonist reverses memory deficits in mouse models of Alzheimer's disease

Hyeon Jeong Kim[1], Haelee Kim[2], Jaeyoung Song[2], Jun Young Hong[3,4], Elijah Hwejin Lee[1,5], Ashwini M. Londhe[1,5], Ji Won Choi[1], Sun Jun Park[6], Eunseok Oh[3], Heeseok Yoon[2], Hoosang Hwang[3], Dongyup Hahn[3,7], Kyungjin Jung[2], Sugyeong Kwon[2], Tara Man Kadayat[2], Min Jung Ma[2], Jeongmin Joo[2], Jina Kim[2], Jae Hyun Bae[2], Hayoung Hwang[2], Ae Nim Pae[1,5], Sung Jin Cho[6], Jong-Hyun Park[1,5,*], Jungwook Chin[2,6,*], Heonjoong Kang[3,8,*], & Ki Duk Park[1,5,*]

[1]Center for Brain Disorders, Korea Institute of Science and Technology (KIST), Seoul 02792, Republic of Korea

[2]New Drug Development Center, Daegu-Gyeongbuk Medical Innovation Foundation, Daegu 41061, Republic of Korea

[3]Laboratory of Marine Drugs, School of Earth and Environmental Sciences, Seoul National University, NS-80 Seoul 08826, Republic of Korea

[4]Department of Systems Biology, Yonsei University, Seoul 03722, Republic of Korea

[5]Division of Bio-Medical Science & Technology, KIST School, Korea University of Science and Technology, Seoul 02792, Republic of Korea

[6]Cureverse, lnc., H2 building, KIST, Seoul 02792, Republic of Korea

[7]School of Food Science and Biotechnology, Kyungpook National University, Daegu 41566, Republic of Korea

[8]Research Institute of Oceanography, Seoul National University, NS-80, Seoul 08826, Republic of Korea

Table of Contents

1. Supplementary Methods

Synthesis of **a1** --3

Synthesis of **b1** --3

Synthesis of **c1** --3

Synthesis of **1** --4

Synthesis of **3a** – **3b** --4

Synthesis of **4a** – **4b** --6

2. Supplementary Results

Molecular modeling --7

Table S1. Docking energies of the studied compound --8

Figure S1. Binding orientation of **5a**, **5b** and GW501516 inside PPARδ ligand binding site ---9

Figure S2. Selectivity of **5a** for peroxisome proliferator-activated receptor δ (PPARδ) ----10

Figure S3. **5a** suppresses inflammatory responses in primary mouse astrocytes ------------11

Figure S4. **5a** treatment inhibits LPS-induced glial activation --------------------------------13

Figure S5. Protective effect of **5a** against cognitive deficits in a mouse model of scopolamine-induced acute AD ---14

Figure S6. Post-administration of **5a** improves scopolamine-induced cognitive impairment in a dose-dependent manner --15

Figure S7. **5a** reduces tau hyperphosphorylation in the brain of APP/PS1 mice ------------17

Figure S8. **5a** induces transcriptional activation of PPARδ target genes *in vitro* and *in vivo* ---18

Figure S9. **5a** enhances Aβ phagocytic activity of microglia in APP/PS1 mice -------------19

Table S2. CYP inhibition, microsomal stability, and hERG inhibition of **5a** -----------------20

Table S3. Pharmacokinetic (PK) parameters of compound **5a** assessed in rats ---------------21

Table S4. Two-strain Ames mutagenic assessment of **5a** --------------------------------------22

Table S5. Data collection and refinement statistics for human PPARδ ligand-binding domain (LBD) (171 – 441) when complexed with **5a**---23

Table S6. Primers and antibodies used in this study --24

3. ^{1}H- and ^{13}C-NMR spectrum of 5a --25

4. HPLC spectrum of 5a --26

1. Supplementary Methods

4-Trifluoromethyl-selenobenzamide (a1)

A suspension of selenium powder (3.95 g, 50 mmol) in 50 mL of ethanol (EtOH) was prepared. Sodium borohydride ($NaBH_4$) (2.02 g, 53 mmol) was added gradually over 1 h to the selenium suspension under a nitrogen atmosphere. The resulting solution was stirred for an additional 25 min. Pyridine (8 mL) and 4-trifluoromethyl-benzonitrile (11.9 g, 72 mmol) were added, and the solution was heated under reflux while HCl (2 M, 25 mL) was added dropwise over 1 h. The resulting solution was refluxed for an additional 30 min and allowed to cool to room temperature (r.t., 20–25 °C) for 3 h. The precipitated 4-trifluoromethyl-selenobenzamide was filtered and washed with H_2O (10 mL) and EtOH (10 mL). The resulting precipitate was dried and recrystallized into an orange solid (84 % yield). ^{1}H NMR (300 MHz, $CDCl_3$) δ 11.07 (b, 1 H), 10.43 (b, 1 H), 7.99 (d, 2 H, J = 8.5 Hz), 7.77 (d, 2 H, J = 8.3 Hz).

4-Methyl-2-(4-trifluoromethyl-phenyl)-selenazole-5-carboxylic acid methyl ester (b1)

4-Trifluoromethyl-selenobenzamide (2.52 g, 10.0 mmol) was dissolved in tetrahydrofuran (THF, 300 mL) at r.t.. Next, methyl 2-chloroacetoacetate (1.22 mL, 10.0 mmol) was slowly added over 20 min while stirring. After completing the addition, the mixture was stirred again at r.t. for an additional 30 min and then refluxed at 80 °C for 12 h. Once the reaction was complete, the mixture was cooled to r.t..

Subsequently, a 50% aqueous solution of NaOH (15 mL) was added and stirred for 20 min. The resultant organic layer was extracted with ethyl acetate, followed by brine, and dried over anhydrous $MgSO_4$. The solvent was evaporated under reduced pressure, yielding 3.33 g (95% yield) of 4-methyl-2-(4-trifluoromethyl-phenyl)-selenazole-5-carboxylic acid methyl ester as a yellow solid. ^{1}H NMR (300 MHz, $CDCl_3$) δ 8.02 (d, 2 H, J = 8.1 Hz), 7.69 (d, 2 H, J = 8.2 Hz), 3.88 (s, 3 H), 2.79 (s, 3 H).

[4-Methyl-2-(4-trifluoromethyl-phenyl)-selenazol-5-yl]-methanol (c1)

4-Methyl-2-(4-trifluoromethyl-phenyl)-selenazole-5-carboxylic acid methyl ester (2.1 g, 6.0 mmol) was dissolved in anhydrous CH_2Cl_2 (100 mL) under a nitrogen atmosphere and cooled

to -78 °C. Di-isobutyl aluminum hydride (DIBAL-H, 16.6 mL, 1.0 M in hexane solution, 2.5 equiv) was slowly added over 30 min, and the mixture was stirred for an additional 30 min at the same temperature. Subsequently, the temperature was raised to -10 °C and stirred for 30 min. Upon completion of the reaction, excess di-isobutyl aluminum hydride was removed using ethyl acetate.

The resulting residue was then extracted with a mixture of 10% sulfuric acid and ethyl acetate and dried over anhydrous $MgSO_4$. The crude product was purified by flash column chromatography using silica gel and then evaporated under reduced pressure. This process yielded [4-methyl-2-(4-trifluoromethyl-phenyl)-selenazol-5-yl]-methanol as a yellow solid (1.74 g, 94% yield). ^1H NMR (300 MHz, $CDCl_3$) δ 7.96 (d, 2 H, J = 8.1 Hz), 7.65 (d, 2 H, J = 8.2 Hz), 4.88 (d, 2 H, J = 5.3 Hz), 2.45 (s, 3 H).

5-Chloromethyl-4-methyl-2-(4-trifluoromethyl-phenyl)-selenazole (1)

[4-Methyl-2-(4-trifluoromethyl-phenyl)-selenazol-5-yl]-methanol (1.13 g, 3.66 mmol) dissolved in anhydrous CH_2Cl_2 (25 mL) was added to triphenylphosphine (TPP, 1.06 g, 4.03 mmol, 1.2 equiv). Subsequently, N-chlorosuccinimide (717 mg, 4.03 mmol, 1.2 equiv) was slowly added at r.t.. Upon completion of the reaction, the solvent was evaporated under reduced pressure. The TPP oxide formed during the reaction was precipitated by adding 20 mL of hexane/ethyl acetate mixture (v/v = 5/1), followed by filtration and evaporation under reduced pressure. Compound 1 was obtained as a yellow solid in 90% yield (1.07 g). ^1H NMR (300 MHz, CDCl3) δ 7.95 (d, 2 H, J = 8.2 Hz), 7.66 (d, 2 H, J = 8.3 Hz), 4.84 (s, 2 H), 2.48 (s, 3 H).

2-Methyl-4-[4-methyl-2-(4-trifluoromethyl-phenyl)-selenazol-5-ylmethylsulfanyl]-phenol (3a)

Isopropylmagnesium bromide (1.5 mL, 2.0 M in ether solution) was added to a solution of 4-iodo-2-methylphenol (2a) (468 mg, 2.0 mmol) in THF (20 mL) at 0 °C. The mixture was stirred for 10 min under a nitrogen atmosphere and cooled to -78 °C. T-butyllithium (2 mL, 1.7 M solution in heptane) was added dropwise, followed by stirring for 30 min at the same temperature. Next, a solution of sulfur (64 mg, 2.0 mmol) in THF (2 mL) was slowly added, and the reaction mixture was warmed to r.t. for 1 h.

The mixture was cooled back to 0 °C, and a solution of 1 (652 mg, 2.0 mmol) in THF (2 mL) was added. The reaction mixture was stirred at r.t. for an additional 30 min. The reaction was monitored by TLC. Once the reaction was complete, it was quenched with aqueous NH_4Cl (15 mL). The organic layer was separated, and the aqueous layer was extracted with ethyl acetate (2 × 10 mL). The combined extract was washed with water, dried over anhydrous $MgSO_4$, filtered, and evaporated under reduced pressure to obtain the crude product. The crude compound was purified by column chromatography on silica gel with hexane/ethyl acetate (v/v = 3/1) to obtain 3a as a white-yellow solid (705 mg, 82%). ^{1}H NMR (300 MHz, $CDCl_3$) δ 7.91 (d, 2 H, J = 8.1 Hz), 7.63 (d, 2 H, J = 8.0 Hz), 7.21 (m, 1 H), 7.12 (m, 1 H), 6.67 (d, 2 H, J = 8.2 Hz), 4.15 (s, 2 H), 2.19 (s, 3 H), 2.17 (s, 3 H).

2-Methyl-4-[4-methyl-2-(4-trifluoromethyl-phenyl)-selenazol-5-ylmethylselanyl]-phenol (3b)

Isopropylmagnesium bromide (1.5 mL, 2.0 M in ether solution) was added to a solution of 4-iodo-2-methylphenol (2a) (468 mg, 2.0 mmol) in THF (20 mL) at 0 °C. The mixture was stirred for 10 min under a nitrogen atmosphere. After it was cooled to -78 °C, t-butyllithium (2.00 mL, 1.7 M solution in heptane) was added dropwise, and the reaction mixture was stirred for 30 min at the same temperature. A solution of selenium (158 mg, 2.0 mmol) in THF (2 mL) was slowly added, and the mixture was warmed to -20 °C for 2 h. After it was cooled back to 0 °C, a solution of 1 (652 mg, 2.0 mmol) in THF (2 mL) was added, and the mixture was stirred at r.t. for an additional 30 min. The reaction was monitored using TLC. Once the reaction was complete, it was quenched with saturated NH_4Cl (15 mL). The organic layer was separated, and the aqueous layer was extracted with ethyl acetate (2 × 10 mL). The combined extract was washed with water, dried over anhydrous $MgSO_4$, filtered, and evaporated under reduced pressure to obtain the crude product. The crude compound was purified by column chromatography on silica gel with hexane/ethyl acetate (v/v = 3/1) to obtain 3b as a white-yellow solid (773 mg, 82%). ^{1}H NMR (300 MHz, $CDCl_3$) δ 7.91 (d, 2 H, J = 8.1 Hz), 7.63 (d, 2 H, J = 8.2 Hz), 7.22 (m, 1 H), 7.12 (m, 1 H), 6.68 (d, 2 H, J = 8.2 Hz), 4.15 (s, 2 H), 2.19 (s, 3 H), 2.16 (s, 3 H).

{2-Methyl-4-[4-methyl-2-(4-trifluoromethyl-phenyl)-selenazol-5-ylmethylsulfanyl]-

phenoxy}-acetic acid ethyl ester (4a)

K_2CO_3 (346 mg, 2.5 mmol) was added to a solution of 3a (430 mg, 1.0 mmol) in aqueous acetone (5 mL), followed by dropwise addition of ethyl bromoacetate (0.134 mL, 1.2 mmol) at r.t.. The reaction mixture was vigorously stirred for 4 h. After completion, the reaction was verified using TLC. The reaction mixture was poured into water (10 mL) and extracted with ethyl acetate (3 × 35 mL). The combined extract was washed with water, dried over anhydrous $MgSO_4$, filtered, and evaporated under reduced pressure to obtain the crude product. The crude compound was purified by column chromatography on silica gel with hexane/ethyl acetate (v/v = 5/1) to obtain 4a as an ivory solid (480 mg, 93%). ^1H NMR (300 MHz, $CDCl_3$) δ 7.90 (d, 2 H, J = 8.1 Hz), 7.64 (d, 2 H, J = 8.2 Hz), 7.25 (m, 1 H), 7.14 (m, 1 H), 6.67 (d, 2 H, J = 8.2 Hz), 4.61 (s, 2 H), 4.23 (m, 2 H), 4.16 (s, 2 H), 2.24 (s, 3 H), 2.21 (s, 3 H), 1.28 (t, 3 H, J = 3.7 Hz).

{2-Methyl-4-[4-methyl-2-(4-trifluoromethyl-phenyl)-selenazol-5-ylmethylselanyl]-phenoxy}-acetic acid ethyl ester (4b)

K_2CO_3 (346 mg, 2.5 mmol) was added into a solution of 3b (477 mg, 1.0 mmol) dissolved in aqueous acetone (5 mL). Subsequently, ethyl bromoacetate (0.134 mL, 1.2 mmol) was added drop by drop at r.t. The resulting mixture underwent vigorous stirring for 4 h. Upon completion, the reaction progress was monitored using TLC. The mixture was poured into water (10 mL) and extracted with ethyl acetate (3 × 35 mL). The combined extract was washed with water, dried over anhydrous $MgSO_4$, filtered, and evaporated under reduced pressure to yield the crude product. The crude compound was purified using column chromatography on silica gel with hexane/ethyl acetate (v/v = 5/1) to obtain 4b as an ivory solid (523 mg, 93%). ^1H NMR (300 MHz, $CDCl_3$) δ 7.90 (d, 2 H, J = 8.1 Hz), 7.64 (d, 2 H, J = 8.2 Hz), 7.27 (m, 1 H), 7.20 (m, 1 H), 6.68 (d, 2 H, J = 8.2 Hz), 4.61 (s, 2 H), 4.23 (m, 2 H), 4.19 (s, 2 H), 2.23 (s, 3 H), 2.15 (s, 3 H), 1.27 (t, 3 H, J = 3.7 Hz).

2. Supplementary Results

Molecular modeling

Docking study shows that **5a**, **5b**, and GW501516 have shown high –CDOCKER energy in PPARδ compared to PPARα and PPARγ (Table S1). This –CDOCKER Energy data supports the high selectivity of these compounds towards the PPARδ. **5a**, **5b**, and GW501516 have shown very similar interactions inside the PPARδ binding site. An acetic acid moiety of these three compounds has shown three hydrogen bonds with HIS287, TYR437, and HIS413 (Figure S1). Selenazole and phenoxy ring in **5a** formed the pi-sulfur bond with CYS249 and MET417 (Figure S1A). **5b** and GW501516 fail to produce pi-sulfur contact similar to **5a** (Figure S1B-C). Instead, they formed pi-alkyl interactions with CYS249 and no interactions with MET417. Superimposed binding poses of these three compounds inside the PPARδ binding cavity are shown in Figure S1D. The orientation of the five-membered selenazole ring in **5a** is perpendicular to CYS249, favorable for making a pi-sulfur bond. The higher strain energy of **5a** (71.73 kcal/mol) resulted from the slightly different orientation of the selenazol ring leads to reduced –CDOCKER energy (50.26 kcal/mol) compared with **5b** (53.82 kcal/mol) and GW501516 (57.73 kcal/mol), respectively. The docking energies of the studied compounds are shown in Table S1. To justify **5a** binding, we calculated the binding energy of these three compounds. **5a** showed higher binding energy -304.03 kcal/mol than **5b** (-284.08 kcal/mol) and GW501516 (-296.31 kcal/mol). Overall, pi-sulfur and hydrogen bond interactions of **5a** contribute to its potential activity towards PPARδ.

Batista et al. in their PPAR selectivity study suggested that VAL312 and ILE328 in the buried hormone binding pocket play a crucial role in PPARδ selective binding [1]. VAL312 and ILE328 in PPARδ are short-side chain residues compared to PPARα (ILE339, MET355) and PPARγ (MET348, MET364). Similar to Batista et al., we also superimposed the binding pose of **5a** inside PPARδ, PPARγ, and PPARα, respectively (Figure S2). In the **5a**-PPARδ complex (magenta) short residues VAL312 and ILE238 allow fitting molecule inside ligand binding pocket in a conformation that is suitable to form the hydrogen bond with HIS413, TYR437, and HIS287. In contrast, the long side chains of the corresponding residues at the same position do not allow the **5a** to form specific conformation, which will constitute the hydrogen bond with crucial residues in PPARα and PPARγ. A modeling study was performed using Discovery Studio 2017 R2 client software [2]. Protein X-ray crystal structures 4BCR (PPARα) [3], 3TKM (PPARδ) [1], and 5TWO (PPARγ) [4] were selected from the RCSB database for docking study.

Ligand docking was performed using -CDOCKER method implemented in Discovery Studio. Images were prepared using Pymol software (www.pymol.org).

1. Batista FAH, Trivella DBB, Bernardes A, Gratieri J, Oliveira PSL, Figueira ACM, et al. Structural Insights into Human Peroxisome Proliferator Activated Receptor Delta (PPAR-Delta) Selective Ligand Binding. PLoS One. 2012; 7: e33643.
2. Dassault Systèmes BIOVIA, Discovery Studio Modeling Environment, Release 4.5(Version), San Diego: Dassault Systèmes, 2016.
3. Bernardes A, Souza PCT, Muniz JRC, Ricci CG, Ayers SD, Parekh NM, et al. Molecular Mechanism of Peroxisome Proliferator-Activated Receptor α Activation by WY14643: a New Mode of Ligand Recognition and Receptor Stabilization. J Mol Biol. 2013; 425: 2878-93.
4. Yi W, Shi J, Zhao G, Zhou XE, Suino-Powell K, Melcher K, et al. Identification of a novel selective PPARγ ligand with a unique binding mode and improved therapeutic profile in vitro. Sci Rep. 2017; 7: 41487.

Table S1. Docking energies of the studied compounds.

Protein	Compounds	EC_{50} (nM)	-CDOCKER Energy (kcal/mol)	Strain Energy (kcal/mol)	Binding Energy (kcal/mol)
hPPARδ	GW501516	1.2	57.73	36.04	-296.31
	**5a**	0.7	50.26	71.73	-304.03
	**5b**	4.3	53.82	66.04	-284.08
hPPARα	GW501516	300	38.26	30.54	-125.99
	**5a**	>10000	30.34	71.13	-112.87
	**5b**	>10000	30.23	30.23	-104.85
hPPARγ	GW501516	1200	34.31	45.93	-109.36
	**5a**	>10000	27.73	79.08	-99.73
	**5b**	>10000	28.26	93.70	-99.80

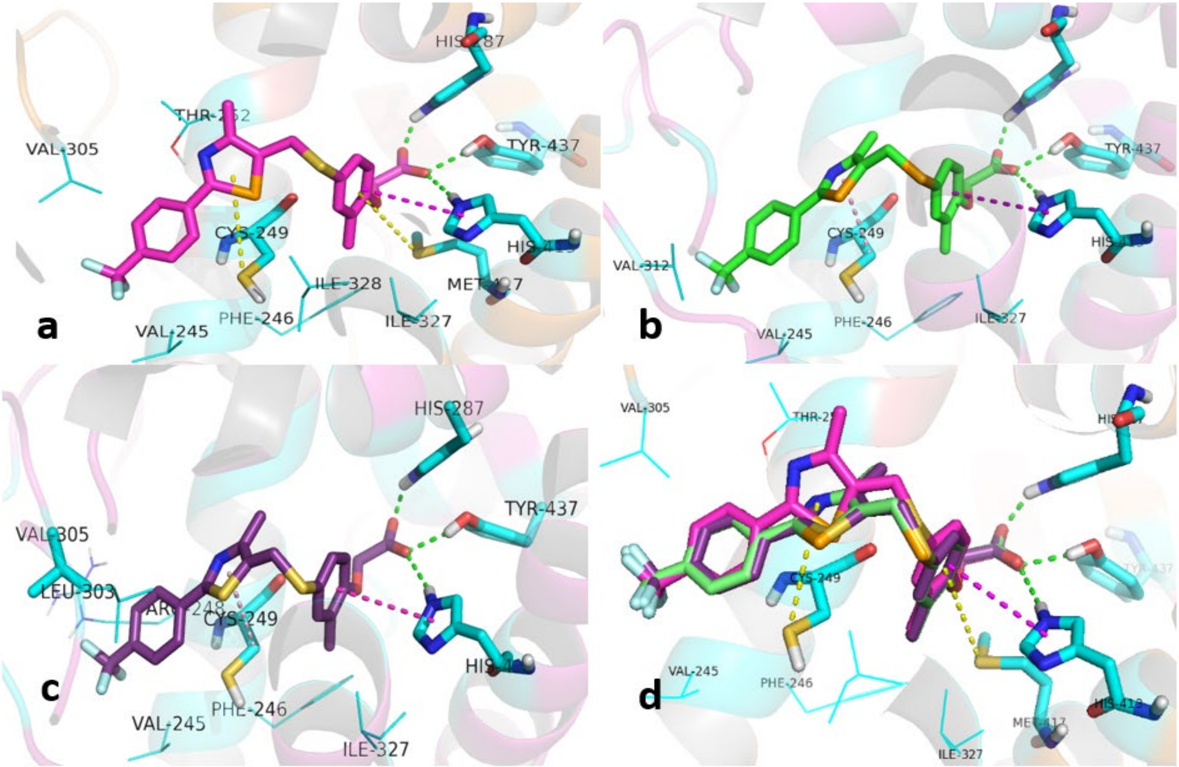

Figure S1. Binding orientation of 5a, 5b and GW501516 inside PPARδ ligand binding site.

Binding orientation of **5a** (a), **5b** (b), and GW501516 (c) inside PPARδ ligand binding site and superimposed orientations of docked ligands (d). Ligand binding site residues were shown in cyan color. Hydrogen bond, pi-pi T-shaped, Pi-sulfur, and pi-alkyl interactions were shown by green, magenta, yellow, and light pink dashes, respectively.

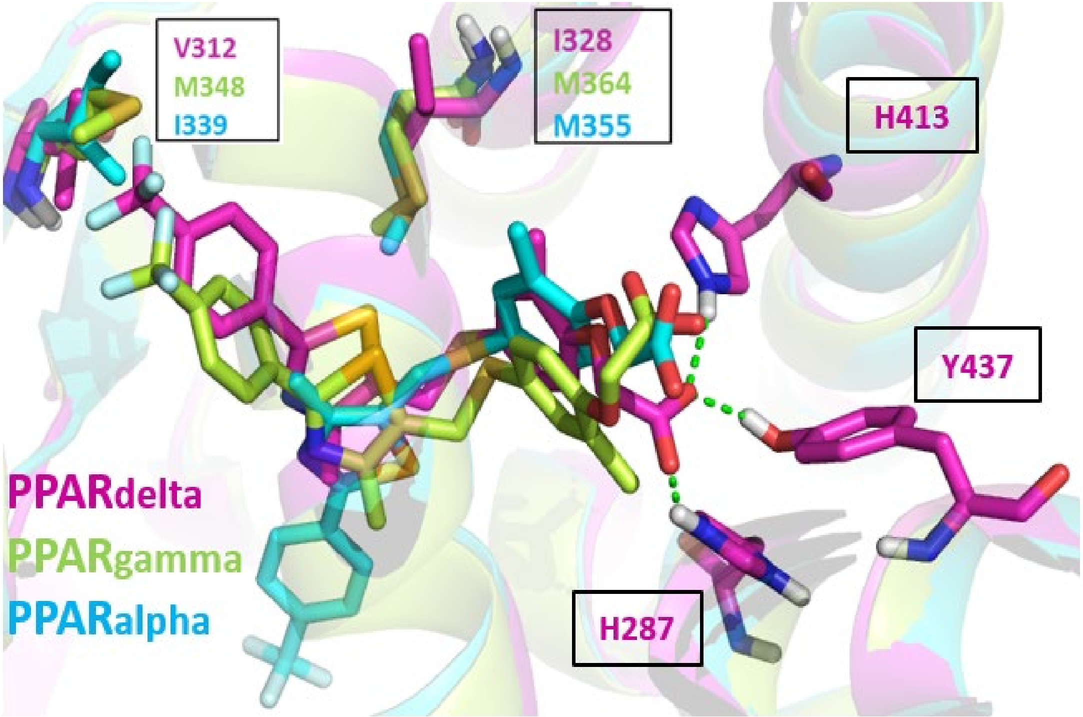

Figure S2. **Selectivity of 5a for peroxisome proliferator-activated receptor δ (PPARδ).**

The superimposed docked pose of **5a** in PPARδ, PPARγ, and PPARα is illustrated. VAL312 and ILE328 residues in PPARδ feature shorter side chains than MET348 and MET364 residues in PPARγ as well as ILE339 and MET355 present in PPARα. Hydrogen bonds formed by **5a** with HIS413, TYR437, and HIS287 are shown by the green dashed lines.

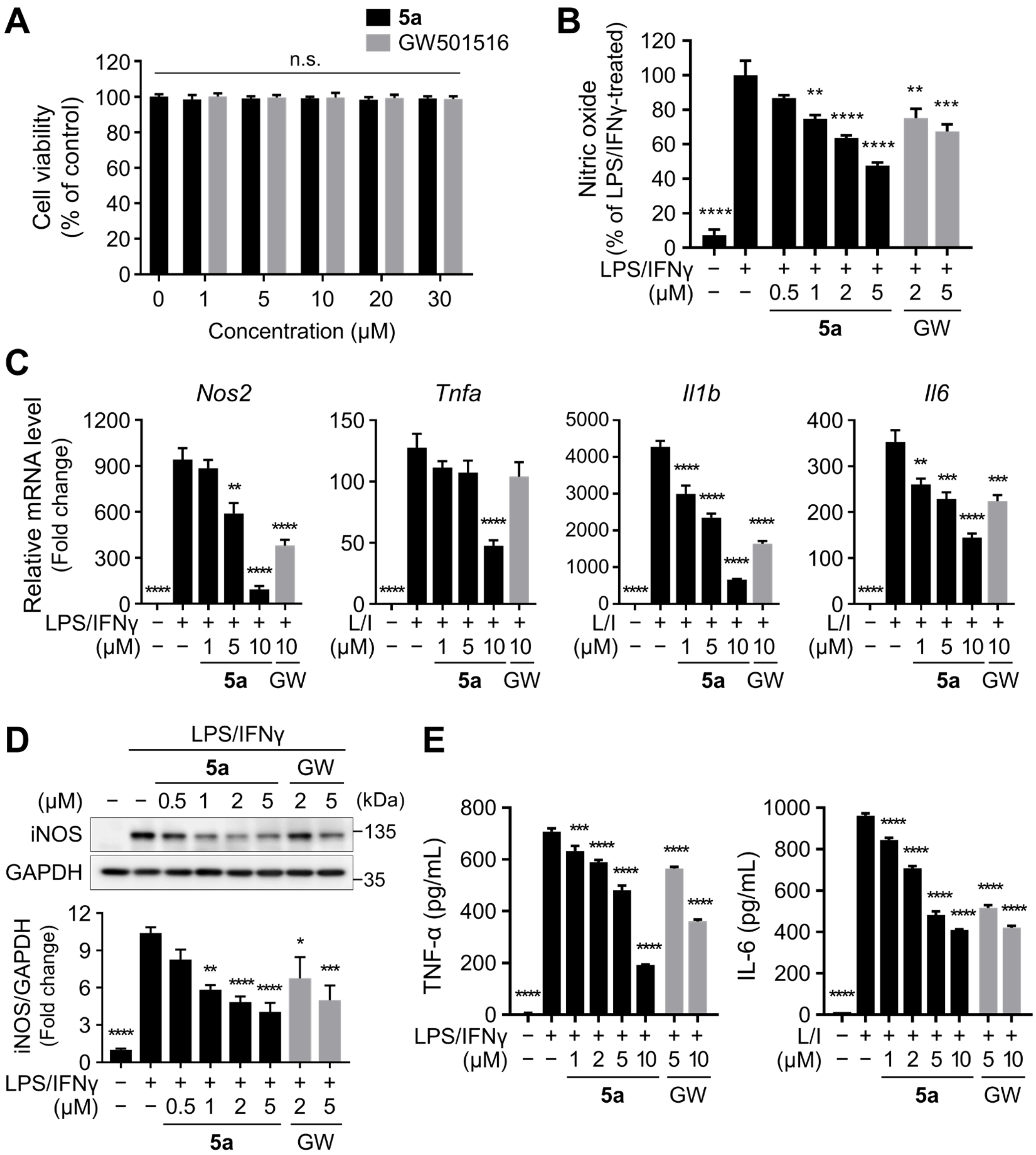

Figure S3. 5a suppresses inflammatory responses in primary mouse astrocytes.

(A) Viability of primary mouse astrocytes after treatment with **5a** or GW501516 for 24 h. n.s., not significant versus vehicle-treated control (one-way ANOVA with Dunnett's test). (**B**) Nitrite levels in the culture medium of primary astrocytes treated with **5a** or GW501516 for 6 h followed by LPS (100 ng/ml)/IFNγ (1 ng/ml) for 42 h. **(C)** qRT-PCR analysis of relative mRNA expression of *Nos2* (*iNos*), *Tnfa*, *Il1b*, and *Il6* in primary astrocytes treated with **5a** or GW501516 for 12 h followed by LPS (10 ng/ml)/IFNγ (1 ng/ml) for 18 h. *Hprt* mRNA levels were used to normalize the expression of each gene. (**D**) Western blot analysis for iNOS in primary astrocytes treated with **5a** or GW501516 for 6 h followed by LPS (100 ng/ml)/IFNγ (1 ng/ml) for 42 h. **(E)** TNF-α and IL-6 levels in the culture medium of primary astrocytes

treated with **5a** or GW501516 for 24 h followed by LPS (10 ng/ml)/IFNγ (1 ng/ml) for 12 h. *P < 0.05, **P < 0.01, ***P < 0.001 and ****P < 0.0001 versus LPS/IFNγ-treated control (one-way ANOVA with Dunnett's test). Data are presented as mean ± SEM. GW: GW501516; L/I: LPS/IFNγ.

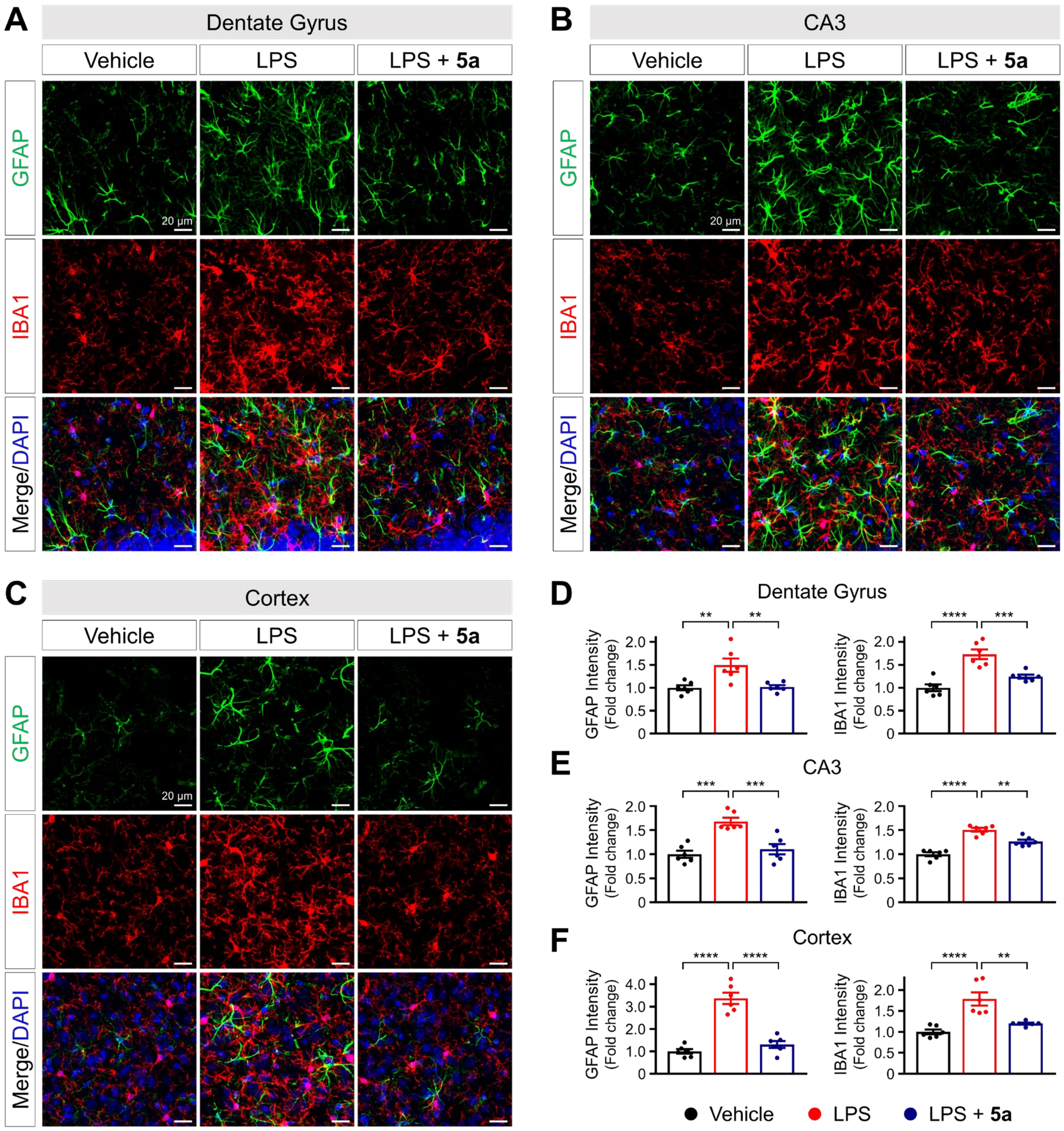

Figure S4. 5a treatment inhibits LPS-induced glial activation.

(A-C) Representative immunofluorescence images showing GFAP (green) and IBA1 (red) in the dentate gyrus **(A)**, CA3 region of the hippocampus **(B)**, and cortex **(C)**. C57BL/6N mice aged 10 weeks were intraperitoneally injected with LPS at 0.3 mg/kg for 7 days and orally administered **5a** at 30 mg/kg for 10 days. **(D-F)** Quantification of immunoreactivity for GFAP (left) and IBA1 (right) in the dentate gyrus **(D)**, CA3 region of the hippocampus **(E)**, and cortex **(F)** (n = 6 brain sections from six mice per group). **$P < 0.01$, ***$P < 0.001$, and ****$P < 0.0001$ (One-way ANOVA with Tukey's test). Data are presented as mean ± SEM.

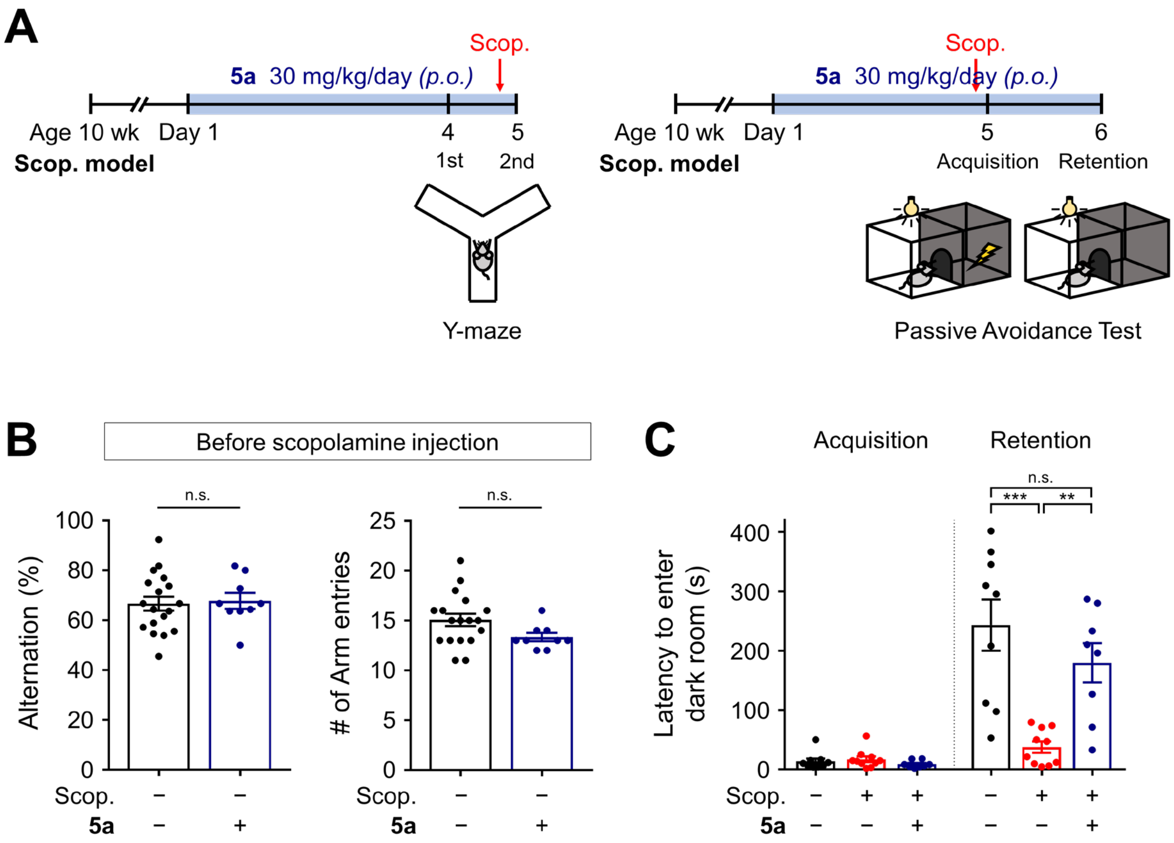

Figure S5. Protective effect of 5a against cognitive deficits in a mouse model of scopolamine-induced acute AD.

(A) Experimental protocol for the Y-maze test (left) and passive avoidance test (right) in vehicle- or scopolamine-treated mice (1 mg/kg, *i.p.*) with or without oral administration of **5a** (30 mg/kg/day for 5 or 6 days) (*n* = 9 mice per group). **(B)** Percentage of spontaneous alternation (left) and number of arm entries (right) in the Y-maze test before scopolamine injection. n.s., not significant (Unpaired two-tailed t-test). **(C)** Latency to enter a dark room in the passive avoidance test. $^{**}P < 0.01$ and $^{***}P < 0.001$; n.s., not significant (One-way ANOVA with Tukey's test). Data are presented as means ± SEM. Scop., scopolamine.

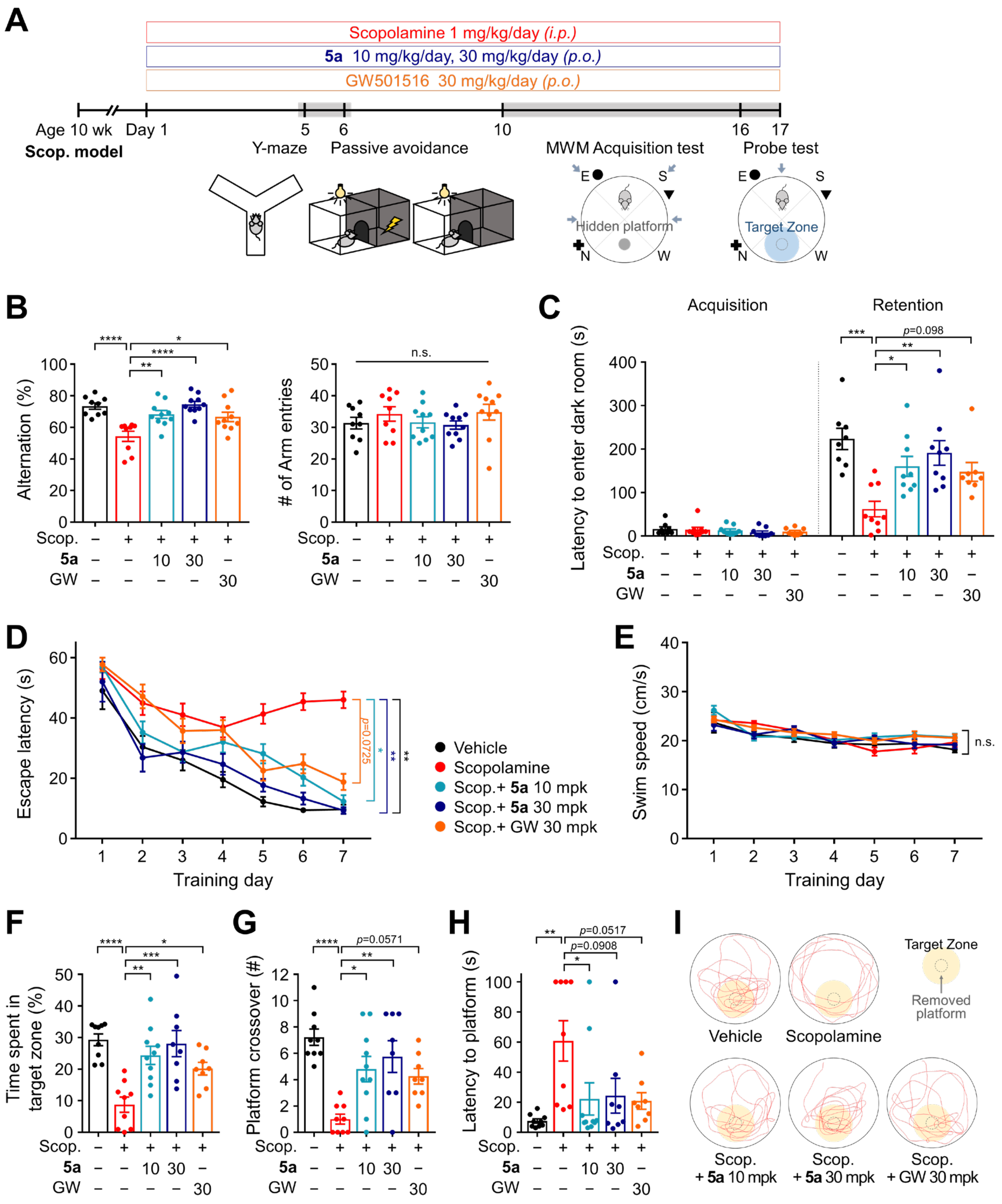

Figure S6. Post-administration of 5a improves scopolamine-induced cognitive impairment in a dose-dependent manner.

(A) Experimental protocol for behavioral tests after oral administration of **5a** (10 or 30 mg/kg/day, *p.o.*) and GW501516 (30 mg/kg/day, *p.o.*) in scopolamine-induced AD mice (1 mg/kg/day, *i.p.*). **(B)** Percentage of spontaneous alternations (left) and number of arm entries (right) in the Y-maze test ($n = 9–10$ mice per group). $*P < 0.05$, $**P < 0.01$ and $****P < 0.0001$;

n.s., not significant (one-way ANOVA with Tukey's test). **(C)** Step-through latency to enter a dark room in the passive avoidance test (*n* = 8–9 mice per group). *$P < 0.05$, **$P < 0.01$ and ***$P < 0.001$ (one-way ANOVA with Tukey's test). **(D-E)** Escape latency to the platform **(D)** and mean swimming speed **(E)** during acquisition tests of the Morris water maze test (*n* = 8–10 mice per group). *$P < 0.05$ and **$P < 0.01$; n.s., not significant (Repeated measures one-way ANOVA with Fisher's LSD (D) or one-way ANOVA with Tukey's test for each day (E)). **(F-H)** Time spent in the target zone **(F)**, the number of passes over the platform **(G)**, and time to reach the platform **(H)** during the probe test of the Morris water maze test (*n* = 8–10 mice per group). *$P < 0.05$, **$P < 0.01$, ***$P < 0.001$ and ****$P < 0.0001$ (one-way ANOVA with Tukey's test). **(I)** Representative track visualization during the probe test. The target zone is indicated by a yellow area centered on the removed platform. Data are presented as mean ± SEM. Scop.: scopolamine; MWM: Morris water maze; GW: GW501516.

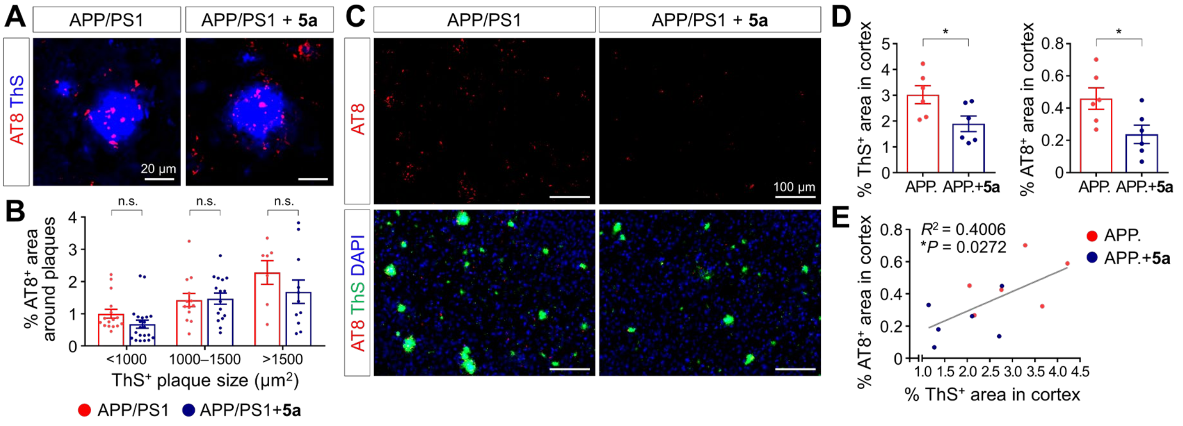

Figure S7. 5a reduces tau hyperphosphorylation in the brain of APP/PS1 mice.

(A) Representative immunofluorescence images showing phospho-tau AT8 (red) around thioflavin S (blue)-positive amyloid plaque in the cortex of 10-month-old APP/PS1 mice treated with vehicle or **5a** (30 mg/kg/day for 10 days, *p.o.*). Scale bar, 20 µm. **(B)** Quantification of AT8-positive area within a 50 µm range centered on thioflavin S-positive plaques (*n* = total 36 plaques from six mice for APP/PS1 and 48 plaques from eight mice for **5a**-treated APP/PS1). n.s., not significant (Student's unpaired t-test). **(C)** Representative immunofluorescence images showing AT8 (red) and thioflavin S (green) in the cortex of APP/PS1 mice treated with vehicle or **5a**. Scale bar, 100 µm. **(D)** Quantification of stained area for thioflavin S (left) and AT8 (right) in the cortex (*n* = 6 brain sections from six mice per group). $*P < 0.05$ (Student's unpaired t-test). **(E)** Positive correlation between tau phosphorylation level and thioflavin S-positive amyloid burden in the cortex of APP/PS1 mice represented in (D). $R^2 = 0.4006$ and $*P = 0.0272$ (two-tailed Pearson’s correlation test). Data are presented as mean ± SEM. ThS: Thioflavin S; APP.: APP/PS1.

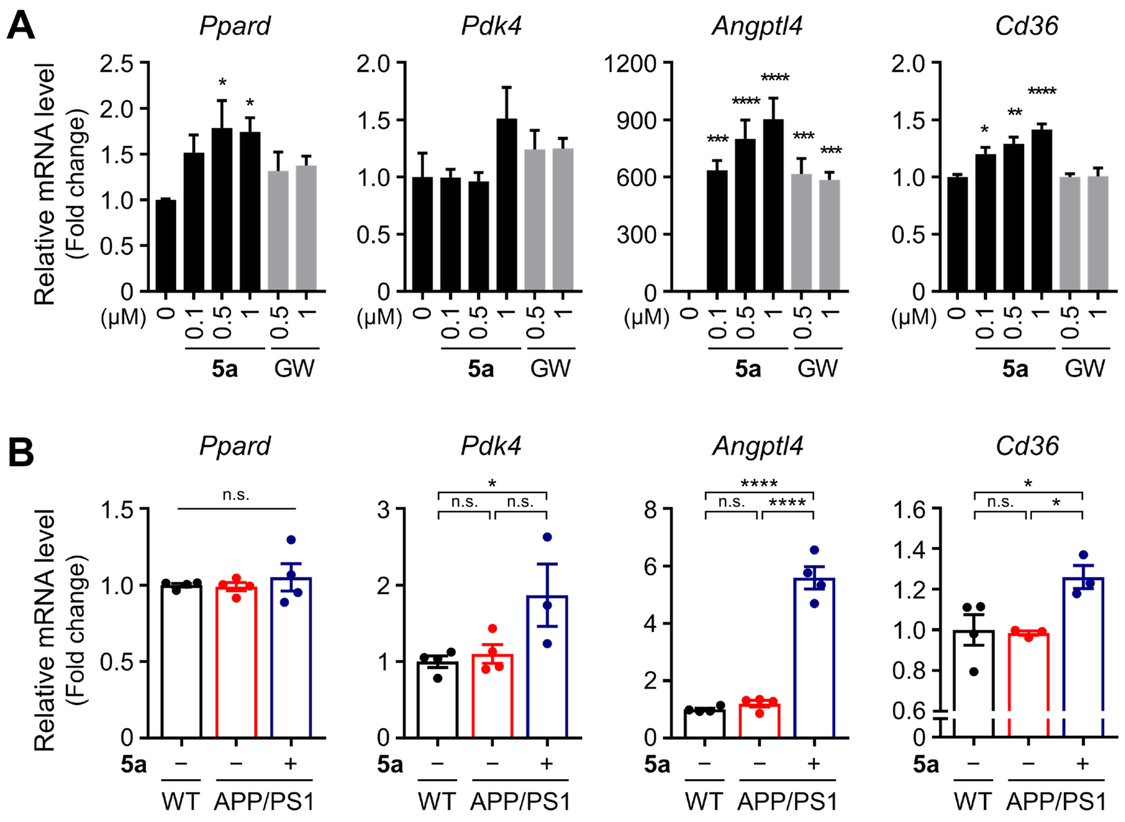

Figure S8. 5a induces transcriptional activation of PPARδ target genes *in vitro* and *in vivo*.

(A-B) qRT-PCR analysis of relative mRNA expression of *Ppard* and its downstream target genes, including *Pdk4*, *Angptl4*, and *Cd36*. **(A)** BV-2 microglia were treated with **5a** or GW501516 for 24 h. *$P < 0.05$, **$P < 0.01$, ***$P < 0.001$ and ****$P < 0.0001$ versus vehicle-treated control (one-way ANOVA with Dunnett's test). **(B)** Cortical homogenates from 10-month-old WT and APP/PS1 mice treated with vehicle or **5a** (30 mg/kg/day for 10 days, *p.o*) were used for analysis (n = 3–4 mice per group). *$P < 0.05$ and ****$P < 0.0001$; n.s., not significant (one-way ANOVA with Tukey's test). *Hprt* mRNA levels were used to normalize the expression of each gene. Data are presented as mean ± SEM. GW: GW501516.

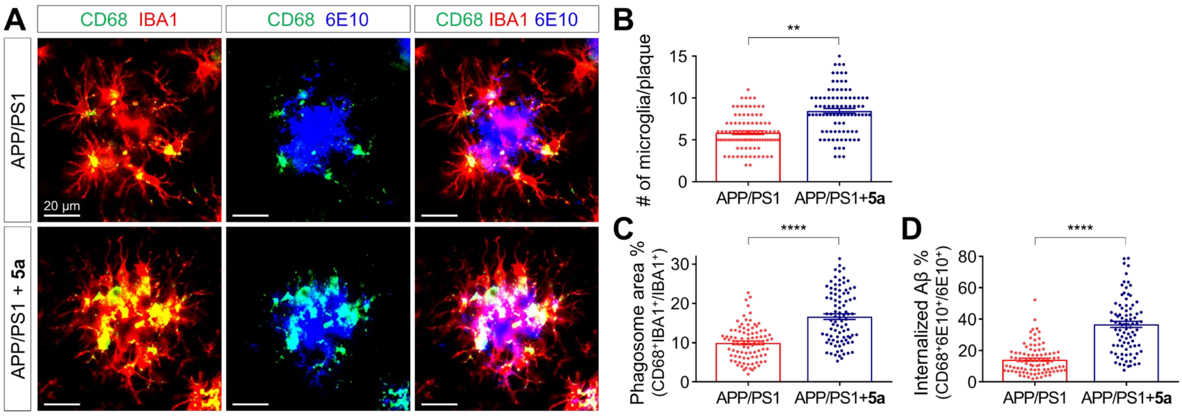

Figure S9. 5a enhances Aβ phagocytic activity of microglia in APP/PS1 mice.

(A) Representative immunofluorescence images showing CD68-positive phagolysosome (green) and IBA1-positive microglia (red) around 6E10-positive Aβ plaque (blue) in the hippocampus of 10-month-old APP/PS1 mice treated with vehicle or **5a** (30 mg/kg/day for 10 days, *p.o.*). Scale bar, 20 µm. **(B-D)** Quantification of the number of microglia **(B)** and colocalized area with CD68 of IBA1-positive microglia **(C)** and 6E10-positive Aβ **(D)** within a 50 µm range centered on amyloid plaques (n = total 90 plaques from eight mice for APP/PS1 and 93 plaques from six mice for **5a**-treated APP/PS1). $^{**}P < 0.01$ and $^{****}P < 0.0001$ (Student's unpaired t-test). Data are presented as mean ± SEM.

Table S2. CYP inhibition, microsomal stability, and hERG inhibition of 5a.

CYP inhibition[a] (% of control activity)					Microsomal stability[b] (% of remaining compound)				hERG inhibition[c] (IC_{50}, μM)
1A2	2C9	2C19	2D6	3A4	Human	Dog	Rat	Mouse	
78.1	63.6	88.6	92.1	94.9	87.7	91.3	73.3	76.1	9.0

[a]Cytochrome P450 (CYP450) enzyme activity was measured by incubating CYP450 enzyme isotype-specific substrates and liver microsomes in the presence of the test compound (10 μM) for 15 min. Data are expressed as percent of CYP enzyme activity relative to that of the vehicle-treated control.

[b]The test compound was incubated with microsomes for 30 min and the percentage of the remaining compound was measured via liquid chromatography-tandem mass spectrometry (LC-MS/MS).

[c]Human Ether-à-go-go-related gene (hERG) channel binding assay was performed using a predictor hERG fluorescence polarization assay (Invitrogen); IC_{50} values were calculated with non-linear regression fit with four parameters.

[a-c]The results of these three assays were obtained through the *in vitro* ADME/Tox study service of KMEDIhub, a contract research organization (CRO). The reported measurements are the mean of duplicate results. The standard deviations are not shown as the two assays were found to be reproducible on repeated testing.

Table S3. Pharmacokinetic (PK) parameters of compound 5a assessed in rats.

PK parameters						
i.v. **(5 mg/kg)**				***p.o.*** **(10 mg/kg)**		
AUC_{all} (ng*h/mL)	CL (mL/min/kg)	V_{ss} (L/kg)	$t_{1/2}$ (h)	C_{max} (ng/mL)	AUC_{all} (ng*h/mL)	F (BA) (%)
7352.5 ± 586.5	10.2 ± 1.2	2.3 ± 0.1	2.3 ± 0.6	2744.3 ± 1029.0	13331.0 ± 5222.2	90.7

Rats (n = 4) were administered **5a** at 5 mg/kg for *i.v.* and 10 mg/kg for *p.o.*. Parameters were calculated from composite mean plasma concentration–time data. Data are expressed as the mean ± SD. The formulation included ethanol 10%, dimethylsulfoxide (DMSO) 10%, Chremophor EL 10%, water 50%, and PEG400 30%.

AUC: area under the curve; CL: clearance; V_{ss}: apparent volume of distribution; $t_{1/2}$: elimination half-life; C_{max}: maximum concentration of the drug; F (BA): bioavailability; *p.o.*: per os; *i.v.*: intravenous.

Table S4. Two-strain Ames mutagenic assessment of 5a.

Strain[a]	Activity in the absence (-) of S9[b]	Activity in the presence (+) of S9[b]
TA98	Negative (-)	Negative (-)
TA100	Negative (-)	Negative (-)

[a]*Salmonella typhimurium* strains TA98 (*hisD3052* mutation) and TA100 (*hisG46* mutation) were used to detect frameshift mutations and base-pair substitution mutations, respectively.

[b]Aroclor 1254-induced rat liver S9 fraction for exogenous metabolic activation.

Table S5. Data collection and refinement statistics for human PPARδ ligand-binding domain (LBD) (171 – 441) when complexed with 5a.

	**hPPARδ (LBD, 171 – 441) / 5a**
**Diffraction data statistics**	
Wavelength (Å)	0.9796
Space group	P 1 2 1 1
Resolution range (Å)	30.80 - 1.70 (1.76 - 1.70)
Unit-cell parameters	
a, *b*, *c* (Å)	39.77 93.90 96.87
α, β, γ (°)	90.00 97.40 90.00
Total reflections	721671
Unique reflections	76595
Mean I/sigma (I)	32.5 (1.50)
Completeness (%)	98.69 (89.90)
R-merge	0.066 (0.963)
Multiplicity	4.0 (2.7)
**Refinement statistics**	
R_{work}/R_{free}	0.2056 / 0.2299
Number of non-hydrogen atoms	
Protein	4192
Ligand	104
Water	250
B-factor ($Å^2$)	
Protein	36.45
Ligand	39.34
Water	41.02
RMSD deviations	
RMS (Å)	0.006
RMS (°)	0.77
**PDB code**	5Y7X

PDB: Protein Data Bank; RMSD: Root mean square deviation.

Table S6. Primers and antibodies used in this study.

Primers used for qRT-PCR analysis		
**Gene**	**Forward primer (5'→3')**	**Reverse primer (5'→3')**
Nos2	AGACCTCAACAGAGCCCTCA	TCGAAGGTGAGCTGAACGAG
Tnfa	CTCTTCTCATTCCTGCTTGTGGC	GAGAGGGAGGCCATTTGGGA
Il6	ACAACGATGATGCACTTGCAGA	GGTACTCCAGAAGACCAGAGGAAA
Il1b	ACTCAACTGTGAAATGCCACCT	ATGTGCTGCTGCGAGATTTG
Gfap	GCCAAGCACGAAGCTAACGA	CCTGGTAACTGGCCGACTCC
Ppard	CATCCTCACCGGCAAGTCCA	TCTCGTTGTAGGGCGGCAG
Pdk4	GCCACGGTCGAGCATCAAG	CGCCTCCTCGGTCAGAAATC
Angptl4	ATGGCCTTTCCCTGCCCTTC	AACCACCAGCCACCAGAGAG
Cd36	TTAATGGCACAGACGCAGCC	CCGAACACAGCGTAGATAGACC
Hprt	CAGGAGAGAAAGATGTGATTGATA	GCCAACACTGCTGAAACA

Primary antibody			
**Antibody**	**Catalog number**	**Company**	**Application**
iNOS	ab178945	Abcam	WB
p-p65	3033	Cell Signaling Technology	WB
p65	8242	Cell Signaling Technology	WB
HO-1	ADI-SPA-895-F	Enzo Life Sciences	WB
BACE1	5606	Cell Signaling Technology	WB, IF
GFAP	AB5541	Millipore	IF
IBA1	019-19741	Wako	IF
CD86	sc-28347	Santa Cruz Biotechnology	IF
6E10	803001	Biolegend	IF
AT8	MN1020	Invitrogen	IF
CD68	MCA1957GA	Bio-Rad	IF
β-Actin	sc-47778	Santa Cruz Biotechnology	WB
GAPDH	G9545	Sigma-Aldrich	WB

WB: Western blot; IF: immunofluorescence.

3. ^{1}H- and ^{13}C-NMR spectrum of 5a

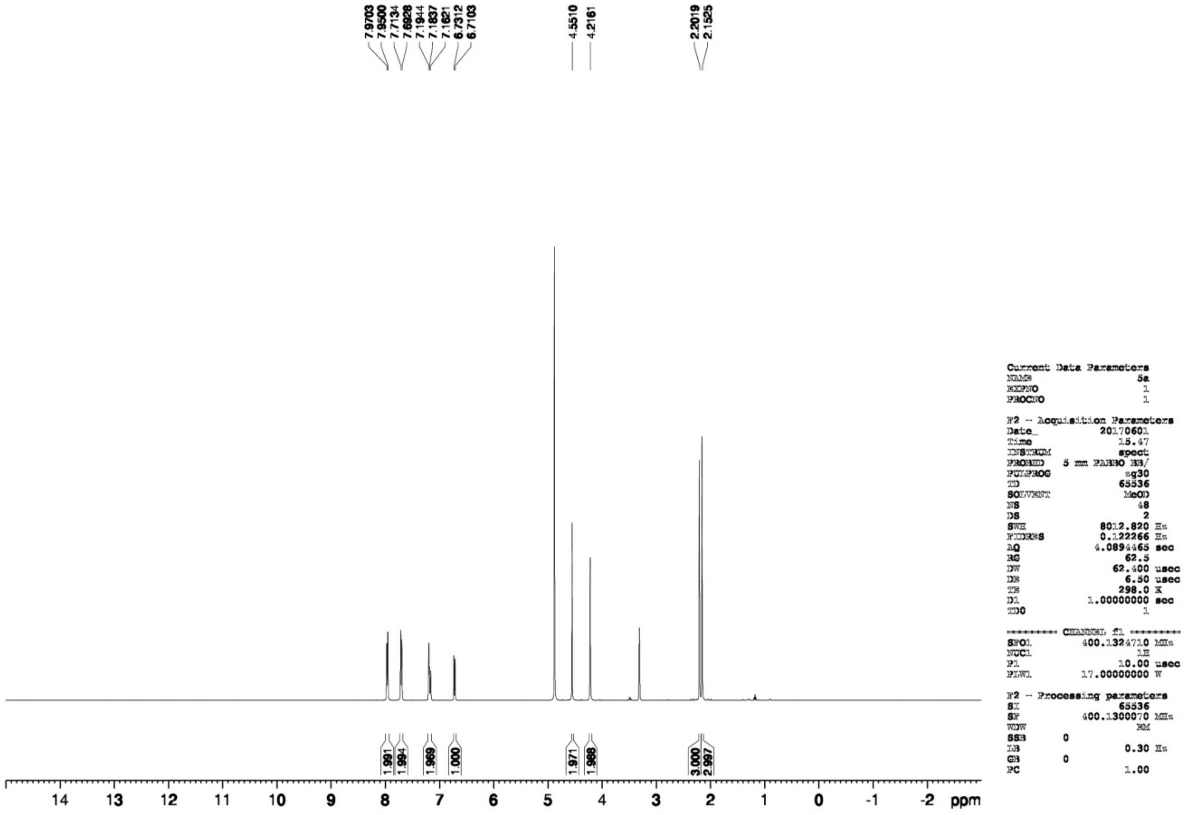

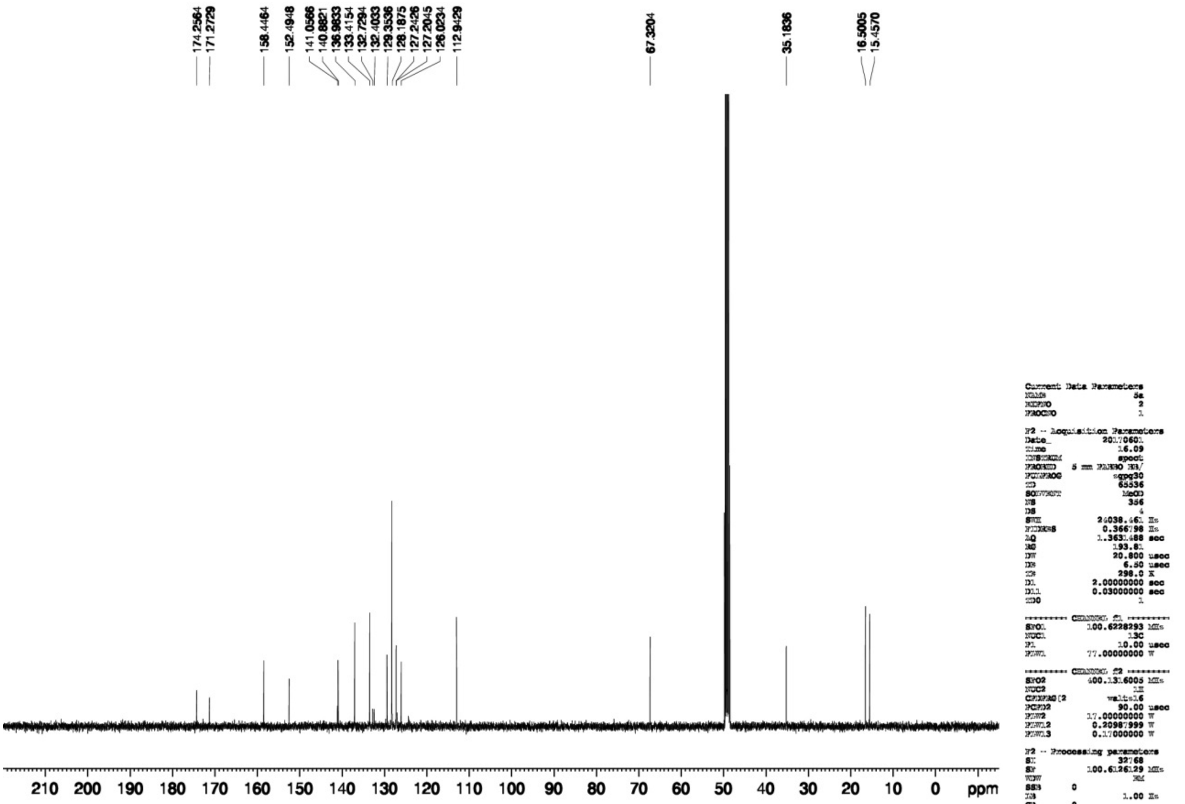

4. HPLC spectrum of 5a (purity 99%)

Operator:n.a. Timebase:U-3000 Sequence:5a Selen

Page 1-1
6/1/2017 5:01 PM

4 5a

Sample Name:	**5a**	*Injection Volume:*	**2.0**
Vial Number:	**BA1**	*Channel:*	**UV_VIS_1**
Sample Type:	**Unknown**	*Wavelength:*	**n.a.**
Control Program:	**0.5ml_min 70%_4.5 min**	*Bandwidth:*	**n.a.**
Quantif. Method:	**cyim**	*Dilution Factor:*	**1.0000**
Recording Time:	**6/1/2017 16:53**	*Sample Weight:*	**1.0000**
Run Time (min):	**4.50**	*Sample Amount:*	**1.0000**

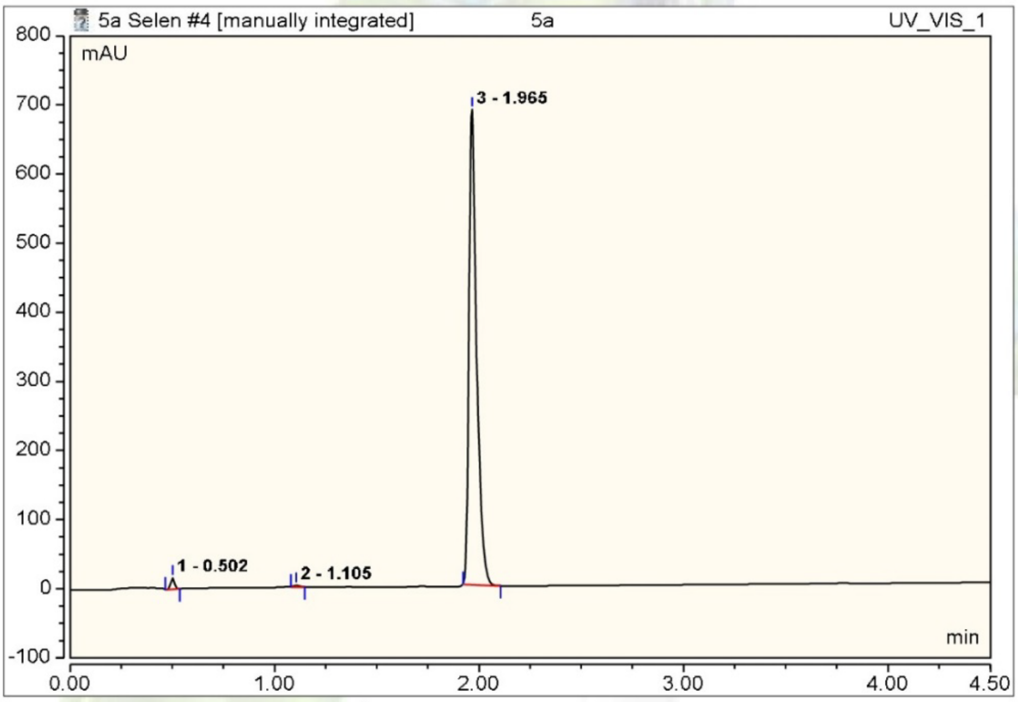

No.	Ret.Time min	Peak Name	Height mAU	Area mAU*min	Rel.Area %	Amount n.a.	Type
1	0.50		16.449	0.320	1.06	n.a.	BMB*
2	1.11		2.313	0.073	0.24	n.a.	BMB*
3	1.97		687.477	29.792	98.70	n.a.	M
**Total:**			706.239	30.186	100.00	0.000	
